# Harvesting random embedding for high-frequency change-point detection in temporal complex systems

**DOI:** 10.1093/nsr/nwab228

**Published:** 2021-12-27

**Authors:** Jia-Wen Hou, Huan-Fei Ma, Dake He, Jie Sun, Qing Nie, Wei Lin

**Affiliations:** Research Institute of Intelligent Complex Systems, Fudan University, Shanghai 200433, China; Centre for Computational Systems Biology, Institute of Science and Technology for Brain-Inspired Intelligence, Fudan University, Shanghai 200433, China; School of Mathematical Sciences, Soochow University, Suzhou 215006, China; Xinhua Hospital Affiliated to Shanghai Jiao Tong University School of Medicine, Shanghai 200092, China; Research Institute of Intelligent Complex Systems, Fudan University, Shanghai 200433, China; School of Mathematical Sciences and Shanghai Center for Mathematical Sciences, Fudan University, Shanghai 200433, China; Department of Mathematics, Department of Developmental and Cell Biology, and NSF-Simons Center for Multiscale Cell Fate Research, University of California, Irvine, CA 92697-3875, USA; Research Institute of Intelligent Complex Systems, Fudan University, Shanghai 200433, China; Centre for Computational Systems Biology, Institute of Science and Technology for Brain-Inspired Intelligence, Fudan University, Shanghai 200433, China; School of Mathematical Sciences and Shanghai Center for Mathematical Sciences, Fudan University, Shanghai 200433, China; Shanghai Key Laboratory for Contemporary Applied Mathematics, LNMS (Fudan University), and LCNBI (Fudan University), Shanghai 200433, China; State Key Laboratory of Medical Neurobiology, and MOE Frontiers Center for Brain Science, Institutes of Brain Science, Fudan University, Shanghai 200032, China

**Keywords:** temporal systems, time series, change-point detection, complex dynamical systems

## Abstract

Recent investigations have revealed that dynamics of complex networks and systems are crucially dependent on the temporal structures. Accurate detection of the time instant at which a system changes its internal structures has become a tremendously significant mission, beneficial to fully understanding the underlying mechanisms of evolving systems, and adequately modeling and predicting the dynamics of the systems as well. In real-world applications, due to a lack of prior knowledge on the explicit equations of evolving systems, an open challenge is how to develop a practical and *model-free* method to achieve the mission based merely on the time-series data recorded from real-world systems. Here, we develop such a model-free approach, named temporal change-point detection (TCD), and integrate both dynamical and statistical methods to address this important challenge in a novel way. The proposed TCD approach, basing on exploitation of spatial information of the observed time series of high dimensions, is able not only to detect the separate change points of the concerned systems without knowing, a priori, any information of the equations of the systems, but also to harvest all the change points emergent in a relatively high-frequency manner, which cannot be directly achieved by using the existing methods and techniques. Practical effectiveness is comprehensively demonstrated using the data from the representative complex dynamics and real-world systems from biology to geology and even to social science.

## INTRODUCTION

The concepts of complex systems and complex networks have made a significant impact in many areas [1–4], such as neuroscience [5], cell biology [6,7], ecosystems [8,9], traffic networks [10] and social sciences [11]. Structures, topologies and networks of complex systems have been found as vital skeletons for the emergence of collective dynamics, diverse phenotypes and advanced functions. While the dominance of the current research approaches assumes static and time-invariant networks in complex systems, it becomes increasingly clear that many networks in practice always change their structure temporally [12–15]. Often, external inputs and fluctuations vary in time and sometimes drastically alter the interactions that connect the individual agents in networks. For example, functional connectivity in the brain network may exert a different pattern caused by drug injections [16]; extinction or blossom of certain species in an ecosystem can lead to different connections of agents in the network [17]; and stock markets are affected by newly adopted domestic and/or international polices as well as influenced by newly added stocks [18].

From observational data gathered from the continuous output of a complex system, is it possible to pinpoint the time at which the system changes its internal structure? Although data sets of time series are regarded as carriers of evolving information on the individual agents and their temporal interacting structures [19–25], they are usually characterized by a tremendously large number of observable agents, high changing frequency of temporal structures and unobservable dynamic deviations from the past. Such intrinsic changes due to a possible change of hidden interacting structures may not be easily separated from other critical changes of the observable dynamics in fixed networks, leading to a major challenge in the accurate detection of change points [26–28].

Two major types of methods have recently been developed for change-point detection. Supervised methods such as the decision tree, the Hidden Markov Model and the Bayesian Inference (BI) [29–31] utilize statistical learning to train classifiers through data sets. Naturally, their performance depends on the size of training data and, in particular, the time series corresponding to each sub-mode of interacting structures needs to be sufficiently long for the method to be accurate. Such long-time data sets are difficult to obtain due to the omnipresence of the high frequency of the structure change. On the other hand, unsupervised methods, such as likelihood-ratio methods, subspace identification and clustering methods, often present an estimation of probability densities before and/or after every change point or group time series into different states [26,32–34]. These unsupervised methods could either be parametric or non-parametric; however, the curse of dimensionality renders it computationally costly to construct an accurate probabilistic model for the joint distribution of many variables in the network [28,35]. Both types of methods assume, to some extent, some prior model structure, lacking the direct incorporation of potential non-linear interactions in the network, which may result in structure changes while only subtle changes in the overall network outputs are observed.

Here, we propose a model-free approach, named temporal change-point detection (TCD), to detect the change points merely on the time-series data sets. The TCD approach involves two essential steps. In the first step, we adapt our recently developed Randomly Distributed Embedding (RDE) framework [23] for generating prediction series. The RDE framework uses delayed and non-delayed coordinates reconstruction theory [36,37] to fully exploit the intrinsic interactions, allowing prediction via integrating information on different combinations of the observable variables. This framework overcomes the difficulty in the shortage of long-time series for dynamics prediction, which is itself a major problem for those broadly used machine-learning methods. Instead of using the RDE for the mere purpose of predicting with short time series, we take into account and quantify the large deviation possibly emergent in prediction series compared to the given time series. Such an emergence of large deviation can indicate an occurrence of essential change in the structure of the network. So, in the second step, to make this indication more quantitatively and practically, we utilize the Bayesian Online Changepoint Detection (BOCD) test—a representative statistical method for change-point detection [38]. To show the practical efficacy of the TCD approach, we apply it to time-series data sets from the representative benchmark models and the real-world systems in which the temporal or time-variant structures are known to make changes. We anticipate that our model-free TCD approach has the potential to be among a set of indispensable tools for identifying fundamental temporal mechanisms when the only access to a complex system is through its observational data.

## A MODEL-FREE APPROACH FOR TEMPORAL CHANGE-POINT DETECTION

For the time points produced by a high-dimensional complex system whose structure is intermittently variant, our TCD approach first utilizes the RDE framework to compute predictions based on these time points in a prescribed sliding window of short length. In principle, when the underlying system undergoes an abrupt structural change, the fidelity of prediction is lost rapidly but recovers to its high level soon after the short window slides through the change point. In order to harvest quantitatively and accurately on this loss of prediction fidelity, viz. a series of change points, along the temporal direction, we develop the TCD approach by integrating the RDE framework, a dynamics-based approach, in a reversal manner with the BOCD test, a representative statistical method of change-point detection. Thus, the TCD approach consists of two major steps, which are designated as follows.

### First step: prediction-series generation using the RDE framework

We use the RDE framework to generate prediction series based merely on the observational time series. For the sake of self-consistence of this work, we hereby review this framework [23]. Let $\{ {{\boldsymbol{x}}( t )} \}$ denote a time series of *m* states generated by an *n*-dimensional dynamical system (abbreviated as $x( t ) \in {\mathbb{R}^n}\ {\rm{for}}\ t = 1,2, \ldots ,m$) and ${x_s}( t )$ be one of the interested target elements of the variable ***x***. According to the generalized embedding theory [36], one can reconstruct the original system not only by a delayed attractor $\mathcal{N} = \{ {{x_s}( t ),{x_s}( {t + \tau } ), \ldots ,{x_s}( {t + ( {E - 1} )\tau } )} \}\ $but also by a non-delay attractor ${\cal M} = \{ {{x_{k1}}( t ),{x_{k2}}( t ), \ldots ,{x_{kE}}( t )} \}$, where ${k_1},{k_2}, \ldots ,{k_E}$ are the indexes randomly selected from the index set {1, 2, …, *n*}. Here, analytically, $E > 2d$ is required and *d* is the box-counting dimension of the system dynamics. Hence, by virtue of the embedding theory, there exists a diffeomorphism between the two attractors, ${\boldsymbol{\Psi }}\!:\ {\cal M} \to \mathcal{N}$. In particular, ${x_s}( {t + \tau } )$ can be expressed component-wise as ${x_s}( {t + \tau } ) = \psi ( {{x_{k1}}( t ),{x_{k2}}( t ), \ldots ,{x_{kE}}( t )} )$. The-refore, a prediction could be made by fitting *ψ* so as to minimize the residue $\|{x_s}( {t + \tau } ) - \psi ( {{x_{k1}}( t ),{x_{k2}}( t ), \ldots ,{x_{kE}}( t )} )\|$, where $\| \cdot \|$ is an appropriately selected norm. Generally, we get different ***ψ*** when different variables are randomly selected as a combination to form a non-delay attractor, and each such embedding reflects the whole-system dynamics from a particular perspective. Normally, the higher the number of different embeddings and mappings that are constructed, the more comprehensive the information extracted from all the variables becomes. Also excluded are the outliers that are produced by those attractors weakly correlated and the whole dynamics cannot be represented. Therefore, we have the following explicit algorithm to make one-step prediction on ${x_s}( {t + \tau } ),$ and calculate the corresponding statistical quantities as follows.

- Randomly select a tuple ${\boldsymbol{k}} = ( {{k_1},{k_2}, \ldots ,{k_E}} )$ from the dimension indexes of the original time series, where $E$ is the number related to the embedding dimension and can be obtained using the standard method [39].
- For this tuple, fit a predictor ${\psi _{\boldsymbol{k}}}$ by minimizing $\|{x_s}( {t + \tau } ) - \psi ( {{x_{k1}}( t ),{x_{k2}}( t ), \ldots ,{x_{kE}}( t )} ){\|_2}$, where the 2-norm is used in this numerical study. Among various fitting methods in the literature, Gaussian Process Regression (GPR) [40] is used in this study.
- Make a one-step prediction as $\hat{x}_s^k( {T + \tau } ) = {\psi _{\boldsymbol{k}}}( {x_{k1}}( t ), {x_{k2}}( t ), \ldots , {x_{kE}}( t ) )$ for a given future time $T + \tau $ where ${\psi _{\boldsymbol{k}}}\ $is obtained using the GPR in the last step. In this study, we simply set $\tau = 1$.
- Repeat Steps 1–3 by randomly picking up another *r* tuples with replacement for *q* times and all the *q* one-step predictions form a prediction set $\{ {\hat{x}_s^k( {T + \tau } )} \}$.
- Exclude the outliers from the prediction set. The outliers are identified by calculating the quartiles of the predictions and the values beyond the upper or lower quartiles are excluded from the prediction set.
- The remaining values in the prediction set form a probability density function $p( {{{\hat{x}}_s}( {T + \tau } )} )$ by using the method of kernel-density estimation [41].
- Set the final prediction by calculating the expectation of the above density function, ${\rm{denoted\ by}}\ \mathbb{E}( {{{\hat{x}}_s}( {T + \tau } )} ).{\rm{\ }}$Calculate the standard deviation, Std, of this density function by ${\rm{Std}} = {\mathbb{D}^{1/2}}( {{{\hat{x}}_s}( {T + \tau } )} )$. And the prediction error, Err, at ($T + \tau )$ for testing the data set could also be calculated as a ${\rm{Err}} = \mathbb{E}( {{{\hat{x}}_s}( {T + \tau } )} ) - {x_s}( {T + \tau } )$.

### Second step: BOCD test on prediction series

Once the one-step prediction at the time instant ($T + 1)$ is made using the training data points in between the time window $[ {1,T} ]$, we shift the window forward by a step using the data in $[ {2,T + 1} ]\ $to predict ${x_s}( {T + 2} )$. Repeating this procedure, we can make predictions successively on the interested target element ${x_s}( t )$. Simultaneously, we also get the curves of the standard deviation and the prediction error along the axis of time. From the two obtained curves, we aim to detect the points that have abnormal values emergent in the quantities of ${\rm{Std}}$ and/or ${\rm{Err}}$. This actually could be regarded as a problem of statistical change-point detection. Accordingly, we apply the BOCD test on the prediction results (including the standard deviation of the predictions and the prediction error). The BOCD algorithm models the time since the last change point, called the run length. The transition probabilities of the run length, named the change-point posterior, are modeled by calculating the posterior predictive distribution over a new observation using the data so far observed before the change point [38]. Logically, the run length at time $t$, named ${r_t}$, can take binary values:

$$\begin{equation*}
{r_t} = \left\{ \begin{array}{@{}ll@{}} {0,}&\quad{{\rm{if\, the\, change\, point\, appears}}}\\&\quad {\rm{at\, time}}\ t,\\ {\ {r_{t - 1}} + 1,}&\quad{{\rm{otherwise}}.} \end{array}\right.
\end{equation*}$$

Here, the BOCD test aims at estimating the run-length posterior distribution $p({r_t}|{x_{1:t}})$ and the posterior predictive distribution $p({x_{t + 1}},|{x_{1:t}})$ with the Bayesian inference [42]. In the BOCD test, this calculation relies on the assumption of the exponential family. Therefore, once the parameters of the run-length posterior distribution and posterior predictive distribution are updated, the distribution matrix of the run length could be determined. Based on the run length, the change point could be located when the run length drops to zero. With some mathematical assumptions on the distributions of the observational data, we include an analytical estimation on the accuracy of the above change-point detection in the online Supplementary Information (SI). This also illustrates why the change point could be identified with a high probability. More specifically, the above procedure could be summarized into the following steps.

- For the interested target element ${x_s}( t )$, make continuous predictions and construct a set containing these predicted values, denoted by $\{ {{{\hat{x}}_s}\!( t )} \}\ {\rm{with}}\ t \in \{ {1,2, \ldots ,M} \}$.
- Calculate the standard deviation of the predictions $\{ {{\rm{Std}}( t )} \}$ and the prediction error $\{ {{\rm{Err}}( t )} \}$.
- Apply the BOCD test to detect the change point of $\{ {{\rm{Std}}( t )} \}$ or $\{ {{\rm{Err}}( t )} \}$ and calculate the distribution matrix of the run length ${R_{M \times M}}$. Initialize the change-point-detection algorithm with ${t_{{\rm{ini}}}} = 0$ and $i = 1$.
- For the *i*-th row ($i < M - {t_{{\rm{ini}}}}$), find the column in which the maximum value of the ${T_{{\rm{col}}}}$-th column of $R( t )$ exists, denoted by ${T_{{\rm{col}},i}} = {\rm{arg}}\mathop {\max }\limits_{} R( {i,1:i - {t_{{\rm{ini}}}}} )$.
- If ${T_{{\rm{col}},i}} \ne i$, i.e. the run-length probability does not reach its maximum value at the diagonal, then the estimated change point is identified as $CP = i - {T_{{\rm{col}},{\rm{i}}}}$ if the distance between the current $CP$ and the last $CP$ is large enough ($ \ge\! 10\ $time instants). If ${T_{{\rm{col}},i}} = i$, let $i = i + 1$ and repeat Step 4.
- Once a change point is detected, reset the starting point ${t_{{\rm{ini}}}}$ to $i + 1$. Repeat Steps 4–6 until the whole time series are covered and no more change points are found.

## MAIN RESULTS

Here, we apply the above-designed TCD approach, a model-free method, to the data sets produced using several representative physical/biological models and three real-world systems. Also, we compare the TCD approach with the other well-known methods in the literature. All the results fully demonstrate the efficacy and usefulness of the approach.

### Benchmark Model I: coupled Lorenz systems

**Figure 1.**
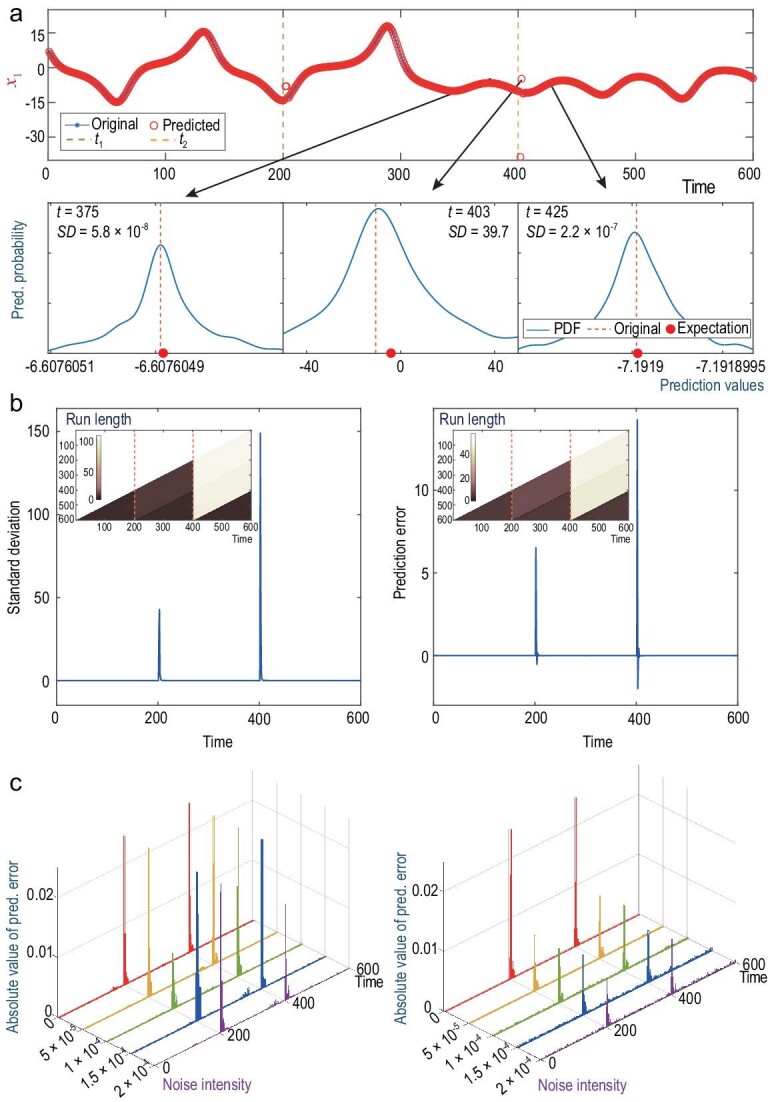
Change-point detection for a model of the coupled Lorenz systems with temporal coupling structures. (a) The observed time series of the variable ${x_1}$ and the one-step predictions, highlighted by circles, using the RDE framework along the axis of time. When the prediction deviates significantly from the observed time series, it suggests a change point in the underlying system dynamics, here occurring at ${t_1} = 200$ and ${t_2} = 400$. Three typical one-step prediction distributions near the second change point are plotted in the lower panel of (a). Here, the distributions are obtained by the RDE framework at each time step, and their standard deviations (SDs) and expectations are computed by the standard method of kernel-density estimation. (b) For all the one-step predictions along the time axis, the SD of the prediction distribution (the left panel) and the prediction error (the right panel) are depicted, respectively. The corresponding BOCD measure values, estimated by calculating the distribution of the run length, are also illustrated. All the run-length-distribution matrices in this article are plotted in a minus log scale with a color bar showing the exponents. (c) The absolute value of the prediction error obtained by using the RDE framework when two types of additive noises with different strengths are applied to the model: the white noise added to the vector fields (the left panel) and the external noise added to the signals of the time series (the right panel), where the outstanding peaks correspond to the change points.

We detect the change points in a representative model: coupled Lorenz systems with temporal coupling structures. First, we study the time-series data generated using the LORENZ60 model, in which 20 individual systems are coupled through an underlying network and each system is described by three variables, denoted by${\rm{\ }}{x_i},{\rm{\ }}{y_i},{z_i}{\rm{\ }}(i = 1,{\rm{\ }}2, \ldots ,{\rm{\ }}20$) (shown in Fig. 1a are the time series of ${x_1}$). The adjacency matrix of the coupling network is prescribed in a temporal manner, i.e. the entries of the matrix change successively over the three constant matrices ${{\boldsymbol{A}}_k}{\rm{\ }}( {k = 1,{\rm{\ }}2,{\rm{\ }}3} )$ and the change occurs at the time indices: ${t_1} = 200{\rm{\ }}$and ${t_2} = 400$, as indicated, respectively, in Fig. 1a (for detailed configurations of the model, refer to Appendix A1 and Fig. S1 in SI). First, we set up a time window of fixed length and apply the RDE framework to the time series inside the window for making one-step predictions. We make the predictions of one and/or some of the interested target time series by sliding the window along the axis of time. Here, we focus on the prediction of the variable ${x_1}$ only. Thus, Fig. 1b show that the two loci are exactly consistent with the corresponding change points that we set in the model, a priori. More precisely, the BOCD measure, estimated by calculating the distribution of the run length, drops down to zero dramatically. At the other points, the BOCD measures keep approximately a linear growth. Clearly, the change points of the coupling structures are successfully detected at a high accuracy using our TCD approach directly, while, since the time series close to the change points do not exhibit instantaneous response to the internal structural change, it is hard to make a precise detection purely using the existent standard techniques of statistics without investigating the dynamics of the system (refer to comparison study presented in the ‘Discussion’ section). Also shown in Fig. 1b, the prediction errors, as well as the standard deviations, sustain at a lower level at other points, but increase drastically right after the change points. So, in addition to the BOCD test, it is sufficient to observe the large deviation emergent in prediction errors or standard deviations for change-point detection in the above coupled Lorenz system.

For the cases in which the coupled systems are perturbed by a certain intensity of noise, the TCD approach still performs well. To demonstrate the ‘robustness’ of this approach against noise, we apply the 15-dimensional LORENZ15a model that is composed of five coupled Lorenz oscillators, where each oscillator is denoted by ${x_i},{\rm{\ }}{y_i},{z_i}{\rm{\ for\ }}i = 1,{\rm{\ }}2, \ldots ,{\rm{\ }}5$, and they are mutually linked by a connection matrix changing successively over the three constant matrices ${{\boldsymbol{B}}_k}{\rm{\ }}( {k = 1,{\rm{\ }}2,{\rm{\ }}3} )$ (refer to Appendix A1 and Fig. S1 in SI). Particularly, two types of additive noises with different intensities are taken into account: the white noise added to the vector fields and the external noise added to the signals of time series. As depicted in Fig. 1c, we are able to accurately locate the outstanding peaks of the prediction error, corresponding to the change points. To be candid, the stronger the intensity of the noisy perturbations, the less efficient the performance of the TCD approach becomes.

We further investigate on the ‘sensitivity’ of the TCD approach with the 15-dimensional LORENZ15b model, where each parameter changes from the default value to a different value at a preset change point (refer to Appendix for the detailed configurations). For each parameter, we estimate the threshold value at which the change point could be just detected and the results are listed in Table S2 and Fig. S9. The results indicate that the TCD approach is able to detect subtle changes. To further reinforce the validation of the efficacy of the approach, we investigate the LORENZ30 model, where the connection matrix ${{\boldsymbol{C}}_2}$ is obtained by rewiring each directional connection from ${x_i}$ to ${x_j}\ ( {i \ne j} )$ in a priori given ${{\boldsymbol{C}}_1}$ with a probability $p$ (refer to Appendix A1). Therefore, $p$, regarded as a parameter, controls the temporal structure of the network, probably leading to different types of dynamical change. In our test, *p* is set at 0.005, 0.01 and 0.25, respectively, and 500 independent simulations are carried out for each *p*. The results, shown in Fig. S12, manifest that the value of the probability can influence the detection accuracy using the TCD approach. For moderate to large $p$, the average number of rewired connections is sufficiently large to influence the dynamics, so that the change point is easier to be detected using the TCD approach. For most cases of small *p*, the rewired connections cannot bring essential difference in dynamics, which renders the TCD approach as losing effectiveness. However, the TCD approach still works for some cases of small *p*. This indeed indicates that the TCD approach could identify those very few but key connections that determine the dynamics of the system.

**Figure 2.**
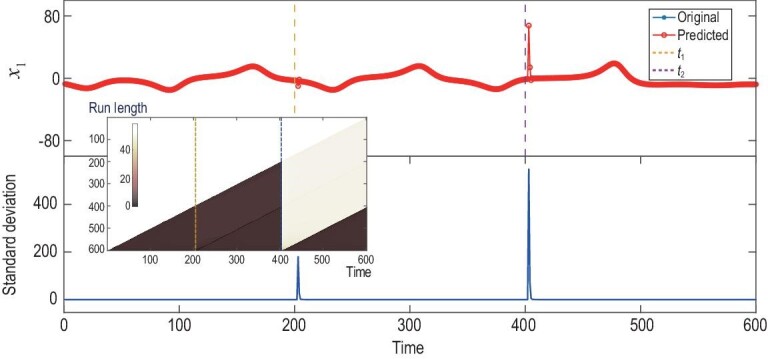
Change-point detection for a model of the coupled Lorenz systems with gradual changes in the network structure. Upper panel: the observed time series of the variable ${x_1}$ and the one-step predictions, highlighted by diamonds, using the RDE framework along the axis of time. When the prediction deviates significantly from the observed time series, it suggests a change point in the underlying system dynamics, here occurring at ${t_1} = 200$ and ${t_2} = 400$. Lower panel: for all the one-step predictions along the time axis, the standard deviations are depicted. Inner panel: the corresponding run-length distributions are illustrated.

Additionally, in real-world systems, dynamical changes may exert a ‘chronic, gradual nature’ instead of a sudden pattern. To validate the capability of the TCD approach for such a situation, we investigate the LORENZ15c model, whose coupling structures ‘change gradually in a linear manner’ (refer to Appendix A1). Shown in the upper panel of Fig. 2 are the time series of ${x_1}$. In this evolving dynamical system, the adjacency matrix that governs the coupling pattern linearly changes from ${{\boldsymbol{B}}_2}$ to ${{\boldsymbol{B}}_3}$, and ${t_1} = 200{\rm{\ }}$and ${t_2} = 400$ stand, respectively, for the beginning and the ending instants of the linear change. Akin to the scenario in the above example of the 60-dimensional Lorenz system, we still successfully detect the above two instants of the linear change period using the TCD method. As is clearly shown in the inner panel of Fig. 2, quantifying the dramatically changed shape of the BOCD measure directly identifies the two instants ${t_1}{\rm{\ }}$and ${t_2}$. It is interesting to note that there is no outstanding fluctuation identified in between the duration (${t_1},$${t_2}$) using our approach. This is because, when the sliding window of the RDE method moves in between the consecutive change points ${t_1}{\rm{\ }}$and ${t_2}$, it is able to treat the gradual change parameter as ‘a new separate variable’ in an embedded system with a sufficiently high dimension. Here, the embedding dimension is set as four, which is larger than the typical dimension of the strange attractor of the uncoupled Lorenz oscillator. However, when the sliding window moves past the change times, the treatment of a new separate variable could be implemented or terminated during a use of the RDE method, which thus can be identified using the BOCD method as shown in Fig. 2.

### Benchmark Model II: biochemical oscillator

**Figure 3.**
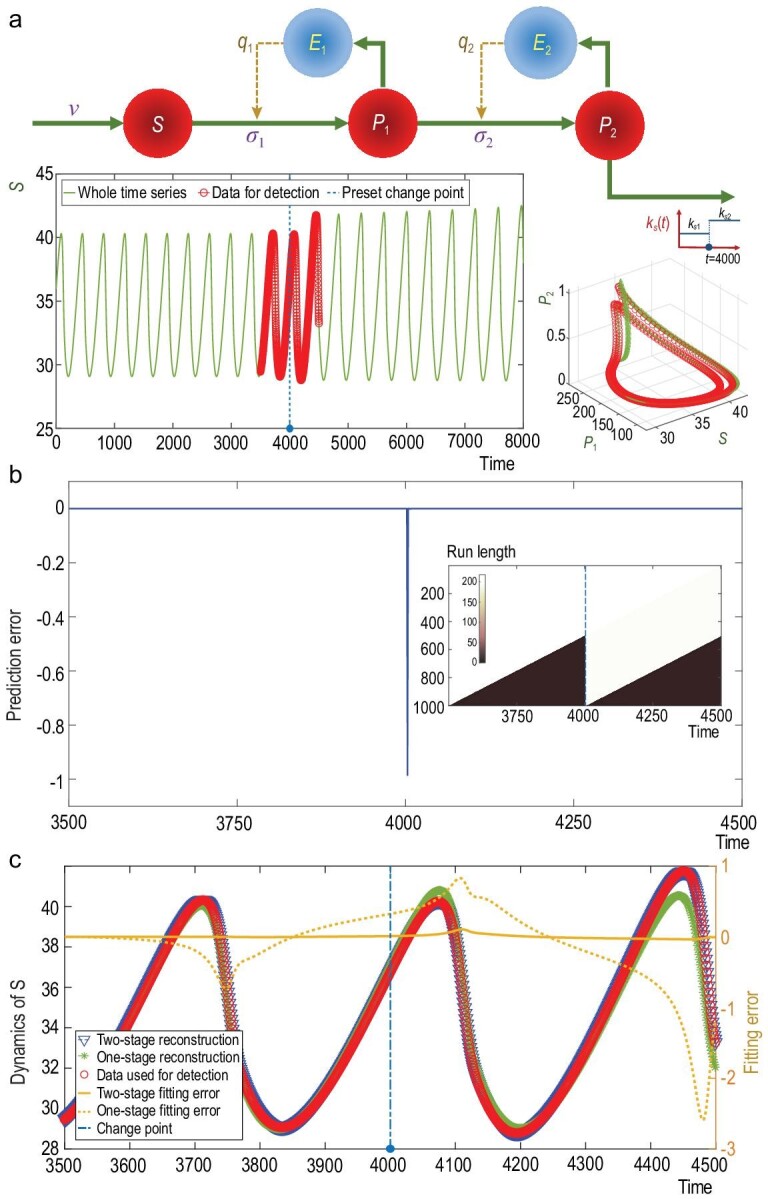
Change-point detection in the model of biochemical oscillation with the parameter undergoing a change. (a) Biochemical oscillations based on the biochemical reaction diagram, where the removal rate ${k_s}$ is a two-stage parameter with a preset change point, $t = 4000.$ The unit of each time point corresponds to 0.25 s, the curves highlighted by the red circles contain the data used for change-point detection and the dynamical behaviors do not alter much immediately after the removal rate changes. (b) The change point detected using our TCD approach is within only five steps after the true change point that was preset (the corresponding distribution of the run length is illustrated in the inner panel). (c) In comparison with the estimated one-stage model, the reconstructed two-stage model has a higher fidelity in restoring the true oscillating dynamics.

Biological organisms and gene regulatory systems, exhibiting complex dynamical behaviors, have been investigated using various types of mathematical dynamical models [7,43,44]. However, due to enormous cost, the number of experiments conducted with high temporal resolution is limited. Often, the regulatory structures in most biochemical models used in data regressions are fixed with an unalterable structure, resulting in inaccurate dynamics that deviate significantly from the true and unmeasured biological outputs. To illustrate this point, we use a biochemical model of enzyme-catalysed kinetics, initially proposed by Decroly and Goldbeter [44]. This model contains two positive enzyme-catalysed feedback loops (see Fig. 3a and Appendix A2 for a detailed description of the biochemical reaction procedure). In this model, we at first set the removal rates ${k_{s1}} = 1.97$, leading to dynamical oscillation of a limit cycle. We set a change point at $t = 4000$, at which the model parameter is set to ${k_{s2}} = 2.00$ leading to chaotic, aperiodic oscillating dynamics. As shown in Fig. 3a, in spite of the essential difference between the two dynamical oscillations with different oscillating periods, the observed time series itself does not appear to undergo a significant change right after the change point we set, thus posing challenges to detecting the change. In order to use our TCD approach for change-point detection, we took the time-delayed coordinates up to two for each variable in this biochemical model, thus making a nine-dimensional observable system (see the SI for the detailed illustration). As shown in Fig. 3b, a remarkable decrease in the BOCD measure appears within only five steps immediately after the true change point. Additionally, based on this change point numerically detected, we use a two-stage model to estimate respective values for the parameter ${k_s}$. Indeed, for these two stages, we obtain ${\hat{k}_{s1}} = 1.970$ and ${\hat{k}_{s2}} = 1.998$ using our prediction results, respectively, with the residual sums of the squares ${\rm{RMSE}} = 1 \times {10^{ - 3}}$ and $0.12$. However, when we use a one-stage model, estimating ${k_s}$ through the entire time series without considering the change point, we obtain an estimation as ${\hat{k}_s} = 1.976$ with ${\rm{RMSE}} = 7.1$. Such an RMSE is significantly higher than those RMSEs obtained by the two-stage model (see Fig. 3c). Therefore, our model-free TCD approach is beneficial in model-driven analysis, allowing identification of the appropriate model configurations and estimating the time-varying parameters.

### Real-world data sets

In addition to testing on synthetic models and dynamics, we demonstrate the usefulness of our TCD approach to change-point detection in several real-world systems, including the data sets from the complex systems of geology, historical glaciology, finance and brain diseases (refer to SI). In the main text, we list three representative examples.

**Figure 4.**
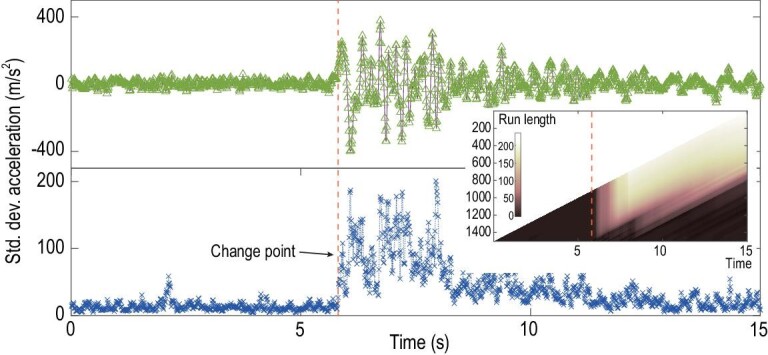
Change-point detection for the earthquake strong-motion data set. Upper panel: the acceleration data of the S60W direction. Lower panel: one change point is detected by measuring the standard deviation of the predictions (black dashed line). Inner panel: the run-length results on the standard deviations of the predictions.

For the first example, we consider a set of earthquake records in Dannevirke (New Zealand) in 1975. The earthquake recorded 5.9 on the Richter scale and had an epicenter ∼15 km south of Dannevirke. Here, we collected its strong-motion data set that contains 100-Hz accelerograph data measured in three orthogonal directions: two directions for movement along the ground and one for vertical movement (see Appendix A3 for more details) [45,46]. The signals from the three directions thus form a 3-dimensional system that could be regarded as hints for complex interactions on the crust of Earth. Before making predictions, we first take the time-delayed coordinates up to five for each direction and thus render the dimension of the system as 18. Then, with a shifting time window containing 50 points (i.e. 0.5 s) as a training set, we predict the kinematics of the S60W direction (illustrated in Fig. 4a, the earthquake begins at $T = - 0.5\ {\rm{s}}$). As is shown in Fig. 4b, the standard deviation of the predictions keeps at a low level most of the time except for a duration starting from ∼6 s. To identify the moment after which the prediction becomes inaccurate, we apply our approach to the standard-deviation series. Our TCD approach successfully reports a corresponding change point at 5.81 s (the black dashed line), which evidently is prior to the signal of the earthquake approaching the first peak (at 5.89 s) and also before it approaches the loci at which the largest motion in one direction occurs (at 6.08 s, corresponding to the lowest valley in the upper panel of Fig. 4). Strikingly, our detection results even surpass those of some of the best recent studies [47], in which the earliest change point detected to date was identified as 5.87 s (almost at the first peak) by applying non-parametric statistical approaches for multivariate piecewise stationary time series. We also evaluate our TCD method using another two strong-motion time series from the same data set and acquire analogous results (refer to Fig. S5).

**Figure 5.**
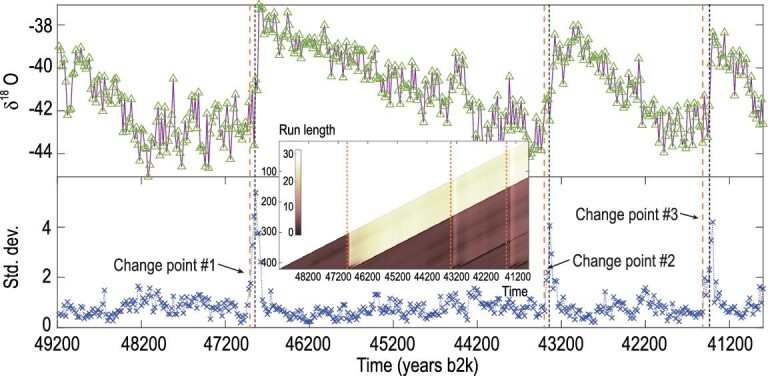
Change-point detection for the Greenland ice-core data set. Here, all ice cores obey the timescale of the Greenland Ice Core Chronology 2005 (GICC05). Upper panel: the δ^18^O concentration (‰) of the NGRIP ice core between 50 and 40 ka. Lower panel: the standard deviation of the predictions obtained by our model-free approach at each time point. Inner panel: three change points (red dashed lines) are highlighted according to the run-length results using the BOCD test. Interestingly, the detected change points are fairly close to but indeed before the estimated DO events that were given in Ref. [50] and depicted in this figure using black dot-dash lines.

As the second example, we consider the data set of the Greenland ice cores. It is known that isotope abundance in ice cores is regarded as a key index used for deducing long-term climate changes in the geological age [48,49]. Here, we consider the δ^18^O data set collected in three drilling programs, called, respectively, NGRIP, GRIP and GISP2, in the ice cores [50,51]. The three drilling sites are regarded as three tips of the iceberg containing complex geographic interactions, where the climate and the hydrologic conditions are considered as external driven forces. The data set consists of the 20-year mean δ^18^O concentration records for the past 104 ka on the GICC05modelext timescale (see part of the records in the upper panel of Fig. 5). We take the time-delayed coordinates up to five for each ice core so as to render the system as having a high dimension (18 dimensions in total) and then use our approach with a window containing 20 time points (i.e. 400 years) as a training set to make the one-step prediction on the NGRIP δ^18^O time series. Due to several historical events of abrupt sea-ice loss, which are called the Dansgaard-Oeschger (DO) events [52], the NGRIP δ^18^O concentration fluctuated correspondingly and remarkably in those event years. We therefore utilize the TCD approach to identify the change points, the DO events, of the abrupt sea-ice loss (refer to Appendix A4). As is interestingly shown in the lower and the inner panels of Fig. 5, the three DO events almost exactly correspond to the three detected change points using our approach. However, the change points directly detected by merely applying the BOCD test to the original data without making predictions using the RDE method are significantly behind the corresponding change points using our TCD approach (see the following comparison study as well as Fig. S6).

**Figure 6.**
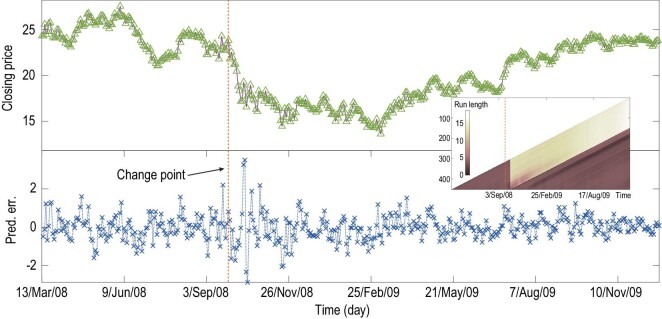
Change-point detection for the data set of the stock market during the 2008 economic crisis. Upper panel: the time series of the closing price of CSCO from March 2008 to December 2009. Lower panel: the prediction errors are calculated and illustrated. Inner panel: the distribution of the run length at each point, which identifies a dynamical change point right after 26 September 2008.

The final example in the main text goes to change-point detection for a particular data set of stock markets. Here, we focus on the time series of the closing prices from three randomly selected companies on each market day during the 2008 global economic crisis that was initially emblematized by the declaration of the insolvency of Lehman Brothers, one of the biggest investment banks, on 15 September 2008. In this example, we consider these three companies as the nodes in a complex financial system regulated by the global economic situation. Although the three companies were not directly linked to Lehman Brothers, they were seriously stricken and suffered great loss from then on. It is of interest to identify how the influence of the declaration of the insolvency of Lehman Brothers dynamically affected every node including these three companies in this complex system. Detection of early signals or change points is beneficial to the understanding of such influence transmission. As such, the time-delay approach is still used to construct an 18-dimensional system (refer to Appendix A5). With our model-free TCD approach equipped with a sliding window of 45 observations, we make predictions on the closing price of Cisco Systems Inc. (CSCO). Figure 6 shows that our approach detects a drastic decrease, right after 26 September 2008, in the run length (see the inner panel) and thus a sudden increase in the prediction errors (see the lower panel). This detected change point reflects an early dynamical change in the financial system that can be regarded as an early consequence of the declaration of the insolvency of Lehman Brothers resulting in the most abrupt decrease in stock prices this century. For the other time duration, the predictability of the closing prices remains relatively reliable. Comparing with the result obtained by using the BOCD test alone on the original time series, our result detects the change point 1 week earlier, suggesting an early-warning function of our approach (see Fig. S7).

### Comparison study

**Figure 7.**
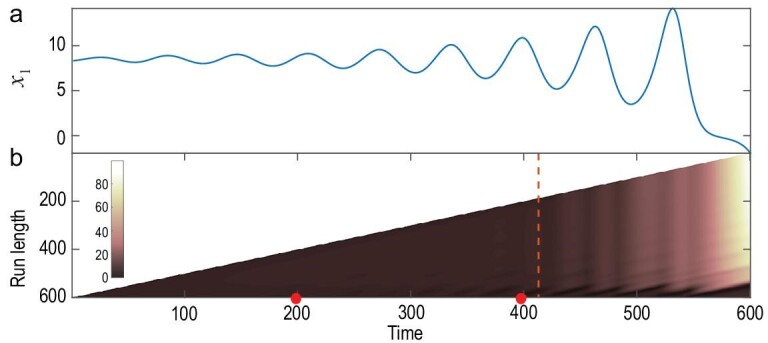
Detection results only using the BOCD test to the original time series produced by the LORENZ15a model with two change points. (a) The time series of the variable ${x_1}( t )$ and the two change points are preset as ${t_1} = 200$ and ${t_2} = 400$. (b) The BOCD test directly implemented on the original time series, where the time instant, as indicated by the red dashed line, is different from the preset change points and is thus a false-positive result.

We compare our model-free approach with several representative statistical methods that are frequently used for change-point detection. First, we apply the BOCD test directly to the original time series (instead of the prediction errors or the standard deviation of the predictions). Here, we consider an example of the LORENZ15a model with two preset change points: ${t_1} = 200$ and$\ {t_2} = 400$ (see Fig. 7a). As shown in Fig. 7b, using the BOCD test on the original time series is unable to either detect the change points or detect a point significantly later than the true change point. We obtain similar results when we use the BOCD test directly on the Greenland data set as well as on the data sets of the stock market during the 2008 economic crisis (see Figs S6 and S7). Furthermore, we compare our TCD approach with the other four widely used methods, viz. the Mann-Kendall test (MK) [53,54], the Cumulative Sum (CUSUM) [55], the Dynamic Programming (DP) [56] and the Pruned Exact Linear Time Test (PELT) [57], using the time points from the LORENZ15a model and from the earthquake strong-motion data set as well. These four methods fail to identify the preset change points accurately for the LORENZ15a model (see results in Fig. S10). Also, the detected change points using these four methods are all behind the marked time points for the strongest motion in the original data set (see results in Fig. S11). As for the case in which the change points are set in a non-uniform manner, we compare our approach with another two typical time-series segmentation methods: Cpdetect and Dynsnap, recently developed in Refs [58,59]. As shown in Fig. S13, a satisfactory detection result is obtained using the TCD approach; nevertheless, both the Cpdetect and Dynsnap algorithms fail to identify any of these two change points set for dynamical systems. All these results therefore suggest that a standard statistical method of change-point detection alone cannot exactly and completely identify those change points that are induced by structural fluctuations in dynamical systems, particularly in non-linear/complex dynamical systems. Our model-free approach however provides a solution to this deficiency when detecting such subtle but key change points.

## DISCUSSION

### Predictability as a prerequisite for measuring unpredictability

Making accurate predictions is one of the focal tasks for data mining using different model-based or model-free approaches [22,60,61]. Our study, contrary to this task, concentrates on measuring the unpredictability and uses the variance in the prediction error to identify temporal instants at which structural changes occur in a complex dynamical system. It should be pointed out that an accurate prediction approach is still a ‘prerequisite’ for what we establish to detect the change points; otherwise, it is baseless to compare those prediction errors obtained by grossly inaccurate prediction methods along the axis of time. For this purpose, we adopt the RDE framework developed recently to realize accurate predictions merely using short-term but high-dimensional time series. The RDE framework produces a probability distribution consisting of various prediction results from different spatial embeddings. Investigating the shape of such distributions also provides a new way of detecting change points because a large standard deviation of the distribution often indicates the existence of weak interactions and/or strong external perturbations to variables and thereby indicates large unpredictability. To measure such an unpredictability more accurately, instead of using a direct visual identification, we articulate a constructive way that combines the RDE framework with the BOCD test—a representative statistical method. In fact, we apply the BOCD test on the prediction errors and/or the standard deviations of the predictions for locating the loci at which the run length falls with a high probability. This test is based not on the original time series, but on the prediction series, which thus integrates all the advantages of the RDE framework into our approach. As shown in all the examples above, our approach has been demonstrated in efficiently identifying change points from systems that are often replete with a moderate strength of noise.

### A short length of window for the training set

**Figure 8.**
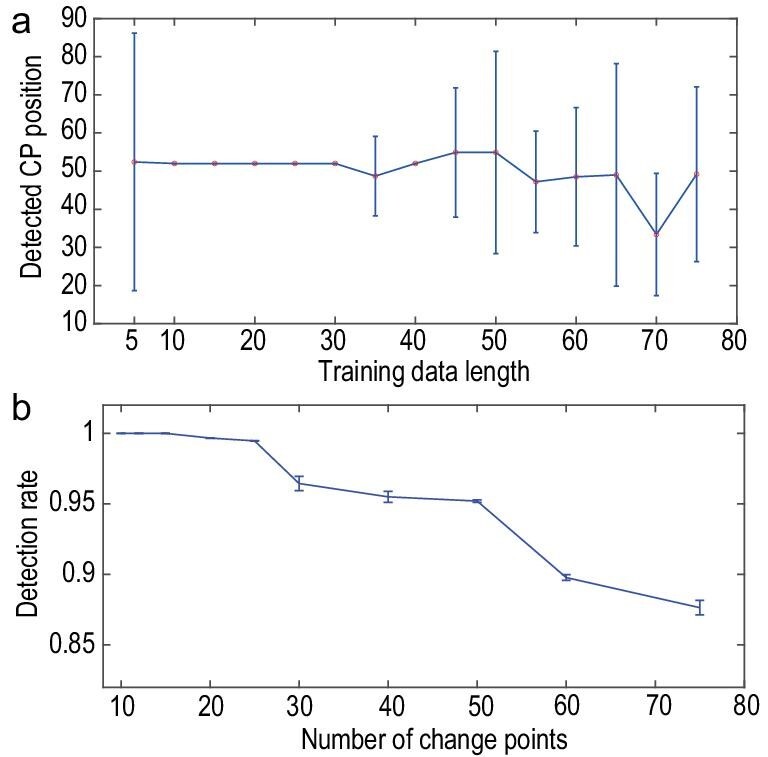
The change-point detection with different lengths of the window for the training data set and with a different number of change points added into the dynamics. (a) The detected change-point position with different lengths of the window, showing that the TCD approach loses its efficacy when its window length becomes either too short (i.e. <10 points for this example) or too long (i.e. >45 points for this example). Here, used as the example is the coupled Lorenz system of 30 dimensions with a change point set as *t* = 50. For every given length of the window, the experiments are repeated 10 times with randomly selected initial values of the coupled Lorenz system. (b) Taken into account is the 15-dimensional coupled Lorenz system with different numbers of changing points. For each produced time series with a given number of change points, the length of the time series is fixed as 700 points. Here, the results in both (a) and (b) are depicted in a manner of ‘mean ± SD’.

One advantage of integrating the RDE framework into our TCD approach is the need for only using short-term data for accurate predictions. Structural changes in real-world systems of high dimension can occur at a high frequency, which makes it challenging for conventional methods that require long-term time series for change-point detection. Our TCD approach, transforming the spatial information into time-course information, is well suited for dealing with change-point detection for dynamical systems switching at a relatively high frequency and using merely time series in a short window. To illustrate this, we test the LORENZ15a model to show that, with a decrease in the window length for the training set, the accuracy of the change-point detection shows a non-monotonic change (see Fig. 8a). As expected, the accuracy drops down when the length of the window becomes long because such a window likely covers the time instant at which or the duration for which the structures of the complex systems change. Also, a shorter training set (a window of <10 time points for the current example) could significantly deteriorate the accurate detection of those preset change points. Similar results are obtained using the Greenland ice-core data set (see Fig. S8). Actually, the prediction accuracy of the RDE method has also been discussed and compared systematically in Ref. [23]. Moreover, as strikingly shown in Fig. 8a, sometimes it is sufficient to use a window covering only 10 time points as the training set to detect the change point. We further test the change-point-detection accuracy by increasing the number of the change points that are added into the dynamics. Here, we use a 15-dimensional coupled Lorenz system but with different switching parameters producing a fixed length of time series (refer to the LORENZ15d model in Appendix A1). As shown in Fig. 8b, although the detection accuracy using our approach decreases gradually as the number of change points increases, the accuracy always sustains a level of >85%. All these demonstrate the effectiveness of our TCD approach in the detection of change points appearing at a relatively high frequency. However, even though our approach is suitable for cases in which change points emerge at a relatively high frequency, it does indeed lose efficacy for those strong and random perturbations continuously injected into the systems. As such, the data produced by these systems become effectively random, making it impossible to distinguish any essential change point from the data.

### Online change-point detection

Generally, change-point-detection problems could be largely divided into two classes: ‘offline’ and ‘online’ detections. Offline detection, a more easily implemented task, uses the time series as a whole and identifies all possible change points at a time. Online detection is a type of real-time task that requires detecting a change point as soon as it occurs or, more practically, demands that the detection be achieved before the next change point appears [62]. In fact, our model-free TCD approach could be used not only for offline detection, but also for online detection in a practical manner. As shown in Fig. 8a, choosing an appropriate short length of the window for training data, the change point in the example can be detected within only two steps after it truly occurs. Furthermore, it is worthwhile mentioning that the integration of the BOCD test into our approach mainly serves as an essential step to detect the occurrence of change points as early and as accurately as possible. This is tremendously valuable for the achievement of online change-point detection.

## CONCLUDING REMARKS

To summarize, we have presented a model-free approach to detect changes in structures in complex dynamical systems. Our TCD approach complements well the existing statistical and/or machine-learning techniques that detect change points based on statistical variances, providing a new set of tools for uncovering hidden dynamical fluctuations induced significantly by temporal structures in the system. We have demonstrated the effectiveness of our approach from several aspects, including cases of change points occurring at a relatively high frequency, online detection of change points in a timely manner and temporal structures appearing in complex dynamical systems of extremely high dimensions (also see change-point detection in brain signals in Appendix A6 and Fig. S13).

Our approach does have some limitations in applications. When the change of the structure in the complex systems is too subtle to arouse the essential change in dynamical behaviors, the TCD approach definitely cannot identify such a change, since our approach crucially relies on the occurrence of dynamical variance (see the sensitivity test in the ‘Benchmark Model I: coupled Lorenz systems’ section). Furthermore, the TCD approach is so far only suitable for time series with a uniform sampling size because using the state space reconstruction always results in such a kind of time series. For non-uniform time series, preprocessing such as an interpolation technique could be introduced. Moreover, our approach is not suitable for dealing with time series perturbed by extremely strong noise because such a case violates the prerequisite for using the TCD approach, i.e. the time points are largely generated by a dynamical system with noise at weak to moderate levels.

Anyway, our TCD approach could be widely used in detecting essential change points in real-world systems with temporal structures—the first step for both data-driven and model-driven research when selecting appropriate piecewise mathematical models. We anticipate that the parameters configured in our TCD approach under the RDE framework and the BOCD test can be further improved, optimized and automated by machine-learning techniques in dealing change-point-detection problems for real-world data.

## Data availability

The source codes for our TCD approach are available at https://github.com/LithiumHou/Temporal-Change-Point-Detection/tree/master.

## Supplementary Material

nwab228_Supplemental_File
