# Supplementary material for: Harvesting random embedding for high-frequency change-point detection in temporal complex systems
Source: Natl Sci Rev. 2021 Dec 27;9(4):nwab228. doi: 10.1093/nsr/nwab228 (PMC9097594; doi:10.1093/nsr/nwab228)
Supplement: nwab228_Supplemental_File [file nwab228_supplemental_file.pdf]

# Supplementary Information on “*Harvesting random embedding for high-frequency change-point detection in temporal complex systems*”

Jia-Wen Hou, Huan-Fei Ma, Dake He, Jie Sun, Qing Nie*, and Wei Lin*

*To whom correspondence should be addressed. E-mails: qnie@uci.edu & wlin@fudan.edu.cn

**Table S1.** Parameters of our TCD approach used in numerical demonstrations for each dataset.

| Model/Dataset | Target series | System dimension | Embedding dimension | Number of embeddings | Window length for training data |
|---|---|---|---|---|---|
| **LORENZ60** | $x_1$ | 60 | 4 | 400 | 20 |
| **LORENZ15a** | $x_1$ | 15 | 4 | 50 | 20 |
| **Length test using LORENZ15a** | $x_1$ | 15 | 4 | 30 | 5～75 |
| **LORENZ15a with noise** | $x_1$ | 15 | 4 | 50 | 20 |
| **LORENZ15b** | $x_1$ | 15 | 4 | 50 | 20 |
| **LORENZ15c** | $x_1$ | 15 | 4 | 50 | 25 |
| **LORENZ15d** | $x_1$ | 15 | 3 | 30 | 15 |
| **LORENZ30** | $x_1$ | 30 | 4 | 200 | 30 |
| **Bionet** | $x$ (the concentration of $\boldsymbol{S}$) | 9 | 3 | 15 | 15 |
| **Earthquake** | S60W acceleration | 18 | 4 | 500 | 50 |
| **Greenland** | GRIP $\delta^{18}$O | 18 | 4 | 60 | 20 |
| **Length test for Greenland** | GRIP $\delta^{18}$O | 18 | 4 | 60 | 10～150 |
| **Stock** | CSCO closing price | 15 | 4 | 300 | 45 |
| **EEG** | Every channel | 18 | 4 | 80 | 25 |

**Table S2.** Sensitivity test for each parameter in the 15-dimensional Lorenz system

| **Parameter** | $\boldsymbol{\sigma}$ | $\boldsymbol{\rho}$ | $\boldsymbol{\beta}$ | $\boldsymbol{C}$ |
|---|---|---|---|---|
| **Default value** | 10 | 28 | 8/3 | 0.1 |
| **Threshold difference** | 0.01 | 0.4 | 0.2 | 0.004 |
| **Relative threshold difference** | 0.001 | 0.143 | 0.075 | 0.040 |
| **Lag(s) between detected and preset at the threshold value** | 2 | 5 | 4 | 2 |

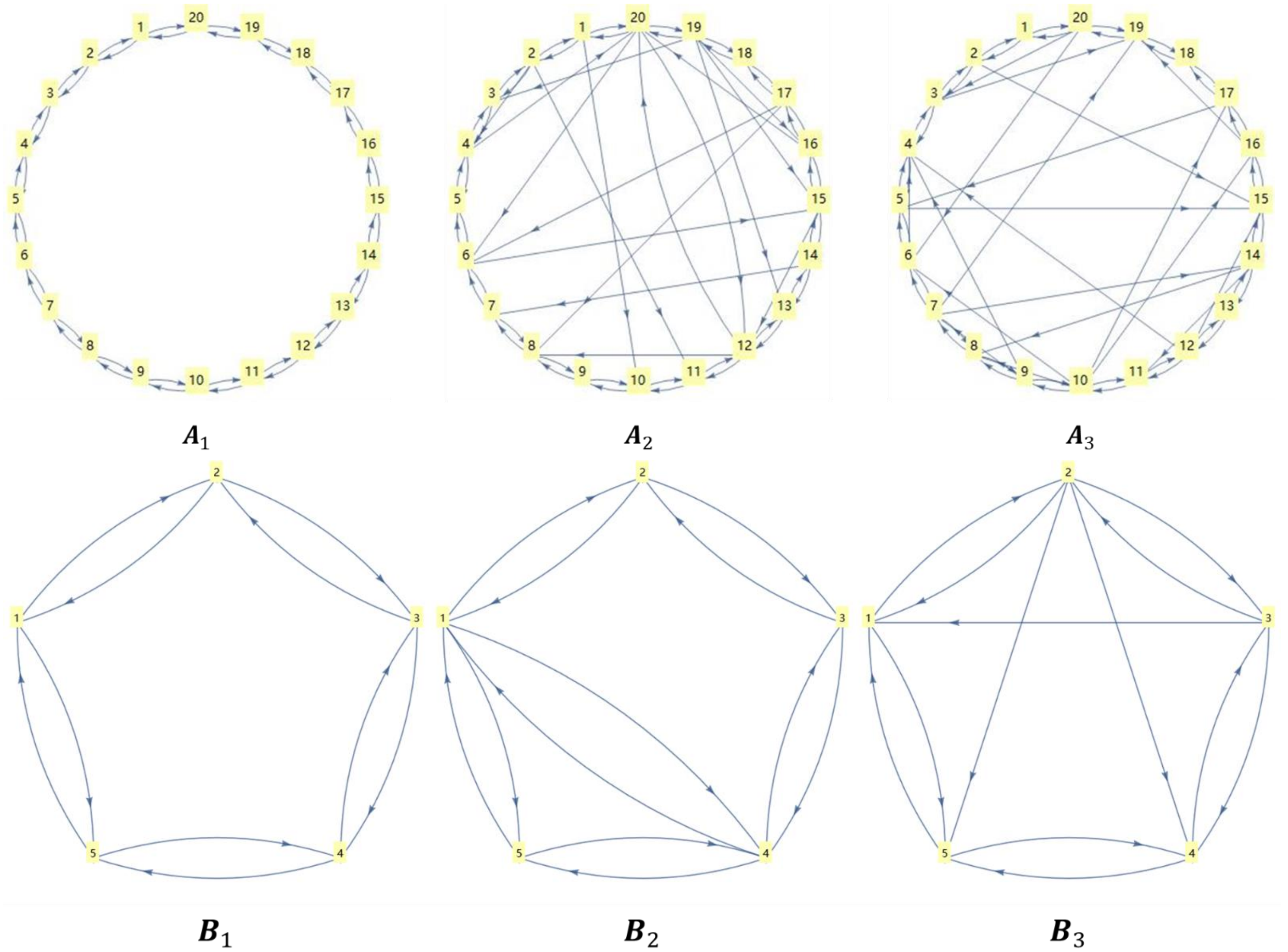


**Figure S1. The temporal adjacent networks for different types of the coupled Lorenz systems. Upper panel**: Three adjacency matrices taking values in the set $\{\boldsymbol{A}_i\}_{i=1,2,3}$ for the 60-dimensional coupled Lorenz systems. **Lower panel**: Three adjacency matrices taking values in the set $\{\boldsymbol{B}_i\}_{i=1,2,3}$ for the 15-dimensional coupled Lorenz systems.

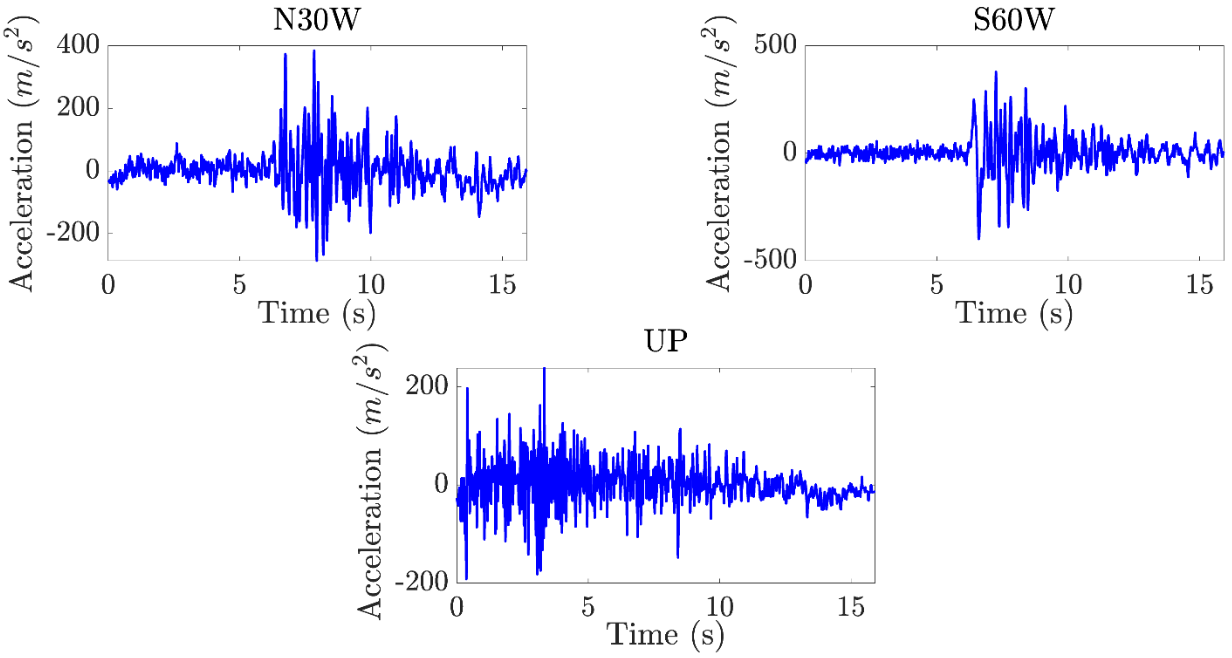


**Figure S2.** The acceleration measures of the three orthogonal directions in the earthquake strong motion dataset.

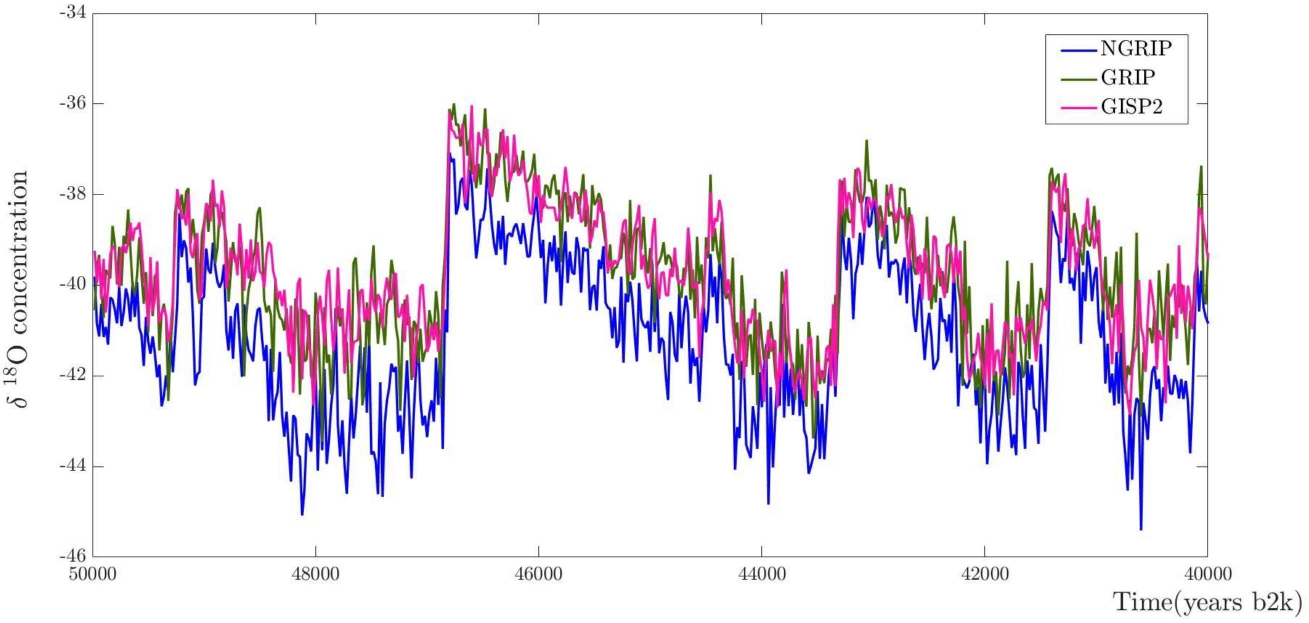


**Figure S3.** The $\delta^{18}$O concentration (‰) of the three ice cores in the Greenland dataset.

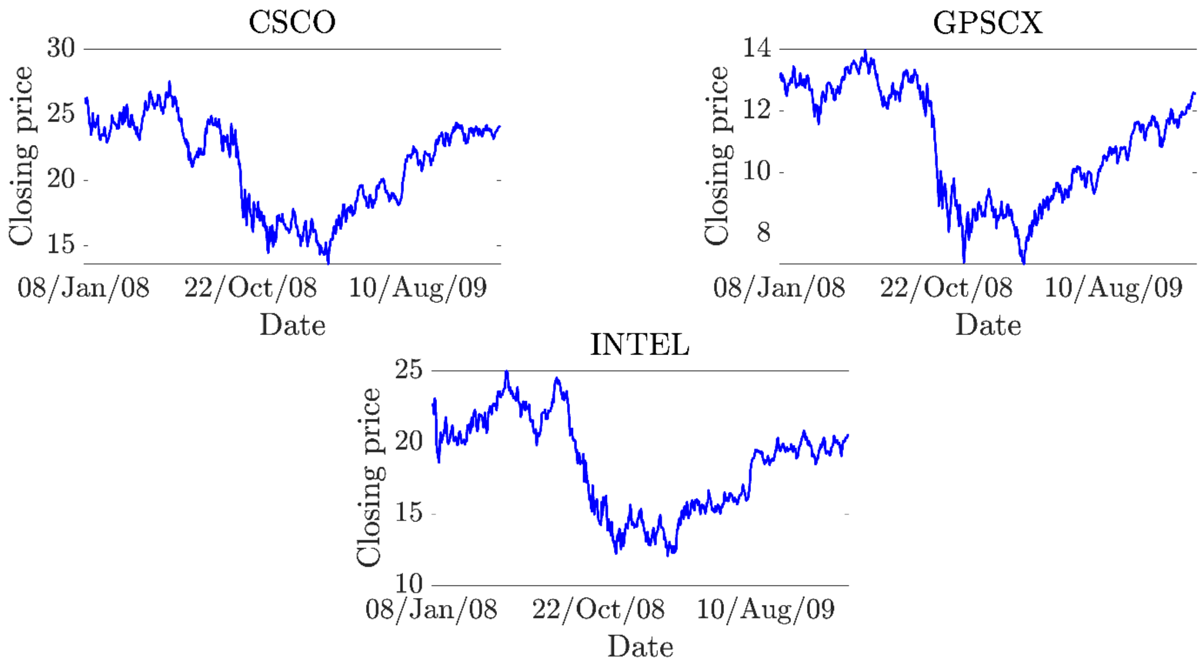


**Figure S4.** The closing price of the three stocks in the financial market dataset.

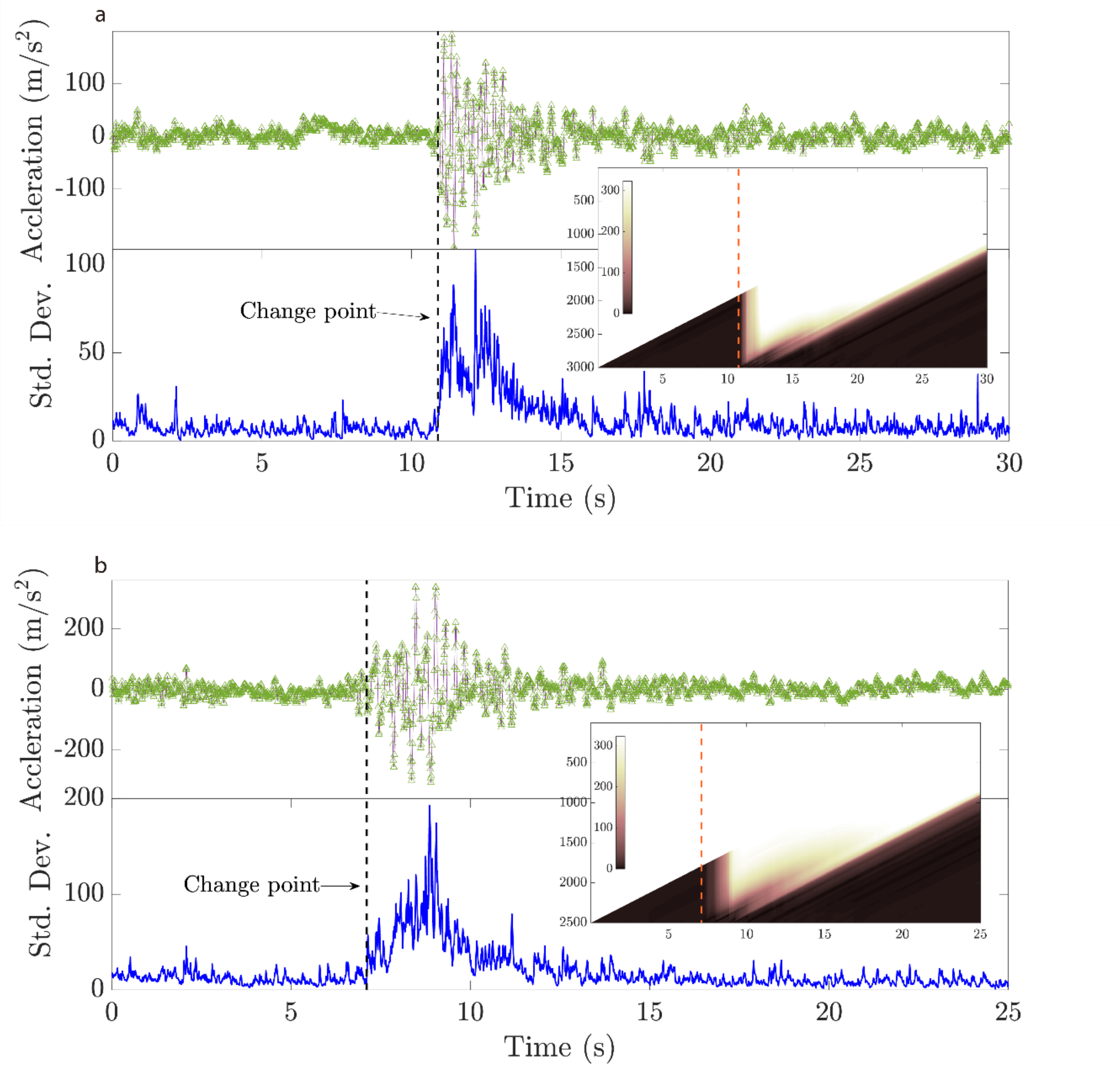


**Figure S5. Change-point detection for another two earthquake strong motion datasets**. **a**, **b.** The results for two different strong motion time series. Here, the change points, identified by using our TCD approach, are highlighted by the black dashed lines.

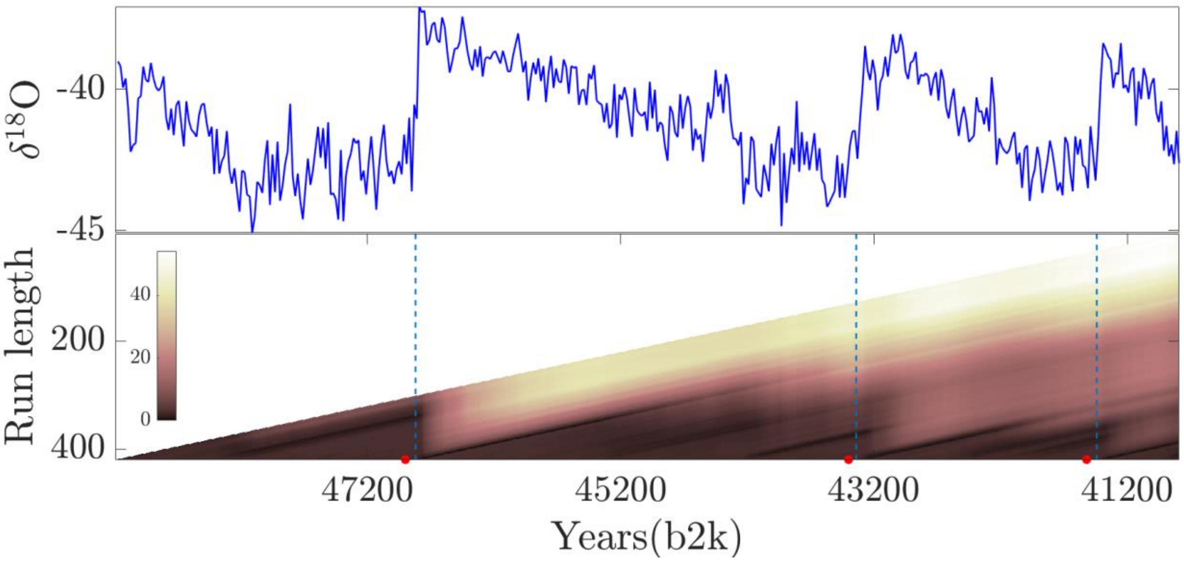


**Figure S6. Comparison study for the Greenland dataset using the original BOCD test.** **Upper panel**: The time series of the GRIP $\delta^{18}$O in the Greenland dataset. **Lower panel**: The BOCD test implemented directly on the original time series. Here, only using the BOCD test to the original time series, three change points are found (illustrated by the blue dashed lines), but all of them are later than the corresponding change points detected using TCD (marked by the red dots).

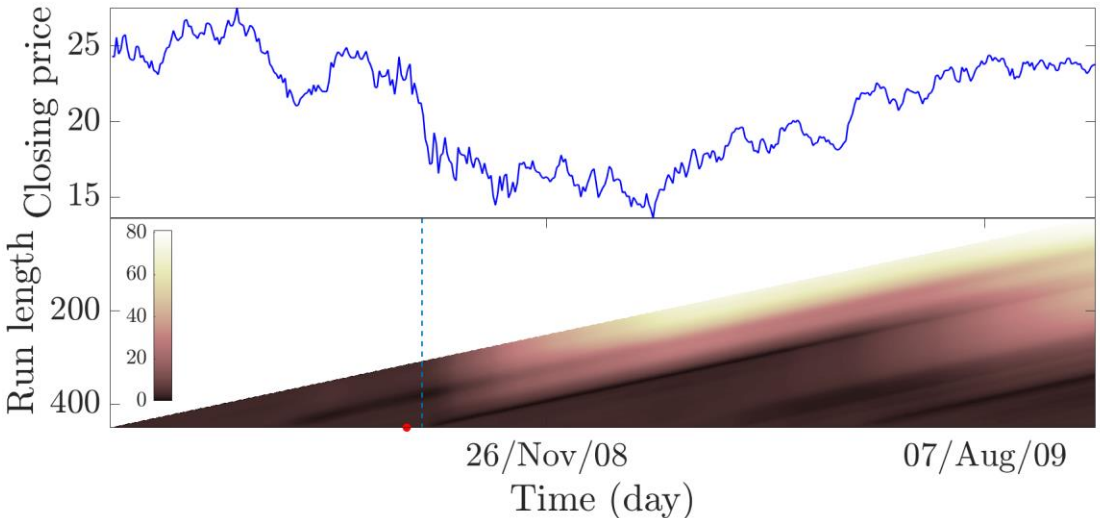


**Figure S7. Comparison study for the stock market dataset during the 2008 economic crisis using the original BOCD test. Upper panel:** The time series of the closing price of the CSCO from March 2008 to December 2009. **Lower panel:** The change point, identified by implementing the BOCD test on the original time series of the closing price (the blue dashed line), is one week later than the dynamical change point identified by our TCD approach (the red dot) in the main text.

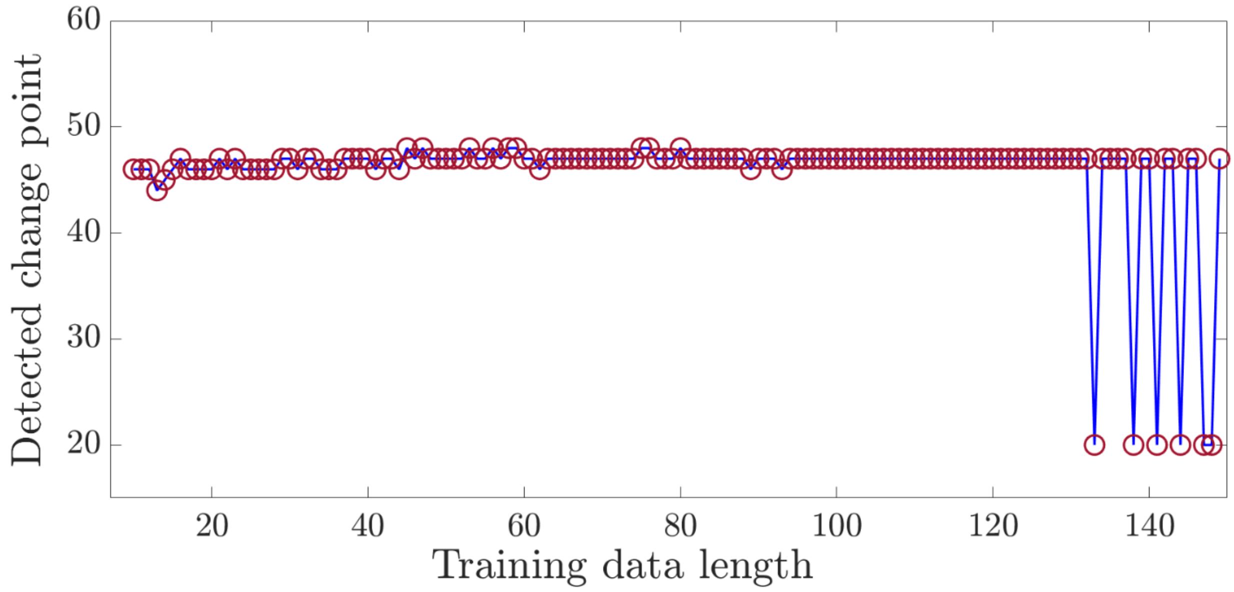


**Figure S8.** Change-point detection with the change of the window length for the Greenland dataset.

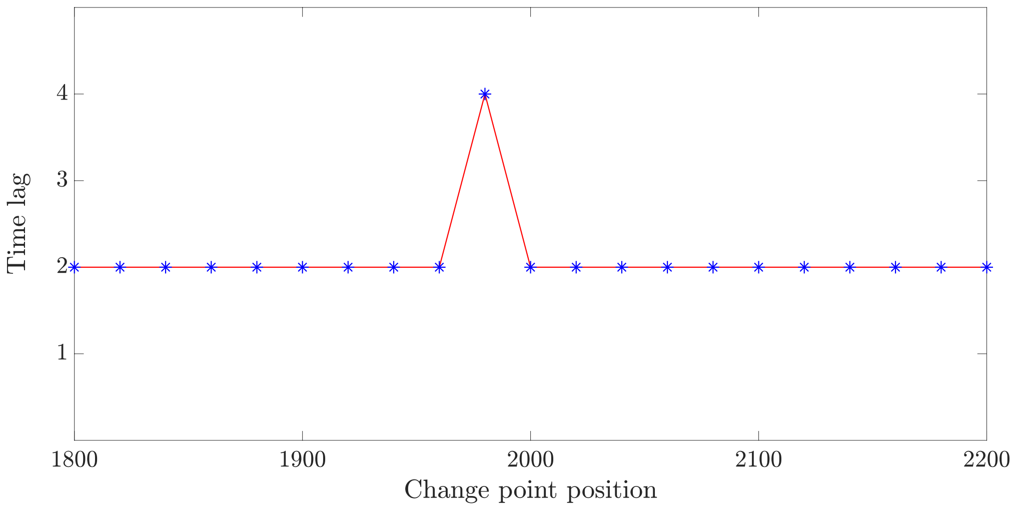


**Figure S9.** The time lag between the identified change point using our TCD approach and the preset change point for the coupled Lorenz system, i.e., the LORENZ15b model (A4).

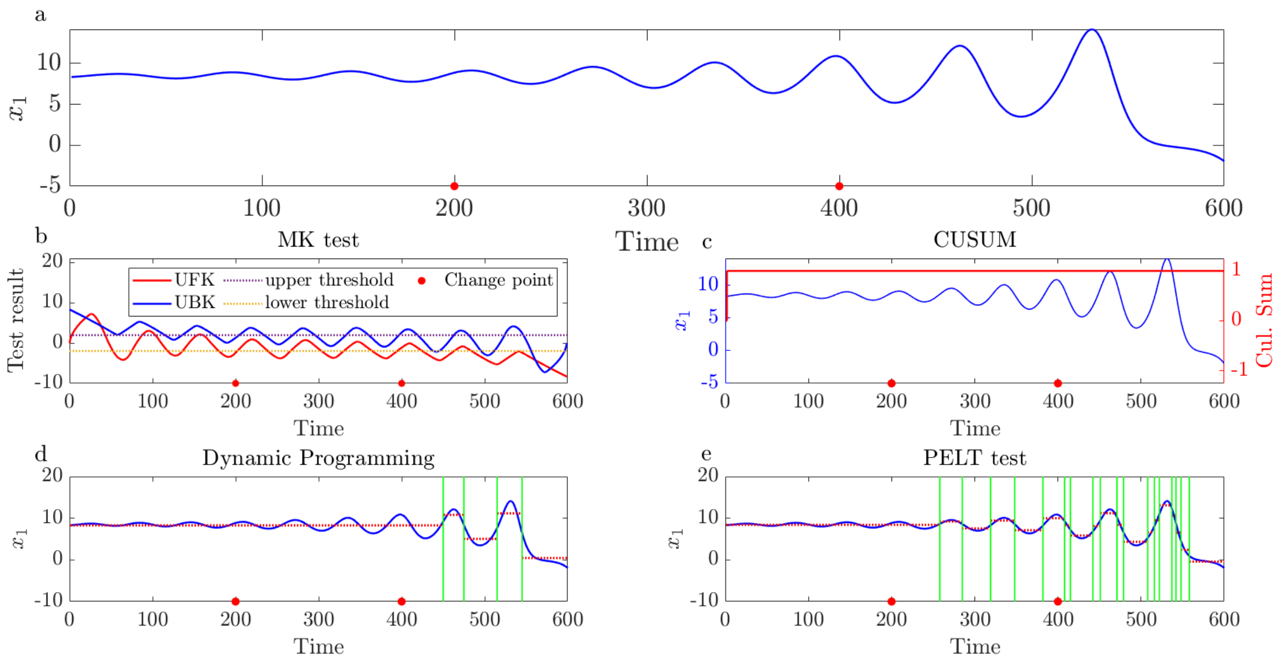


**Figure S10. The detection results, using the four representative statistical methods, respectively, on the LORENZ15a model (A3)**. **a**. The original time series of $x_1$ in the LORENZ15a model, where the change points (red dots) are set at $t = 200$ and $t = 400$. **b**. The results of using the Mann-Kendall test: No significant change points (the intersections of UFK and UBK between the thresholds) are detected. **c**. The results of using the CUSUM: No significant change points (the loci where the Cul. Sum. Measure steps up) are detected. **d, e**. The results of using the Dynamic Programming (DP) and the PELT test, respectively. The detected change points are labelled as the green vertical lines, while the red dotted lines represent the mean values of the time series in each segment divided by these detected change points. Neither the DP nor the PELT detects the preset change points correctly.

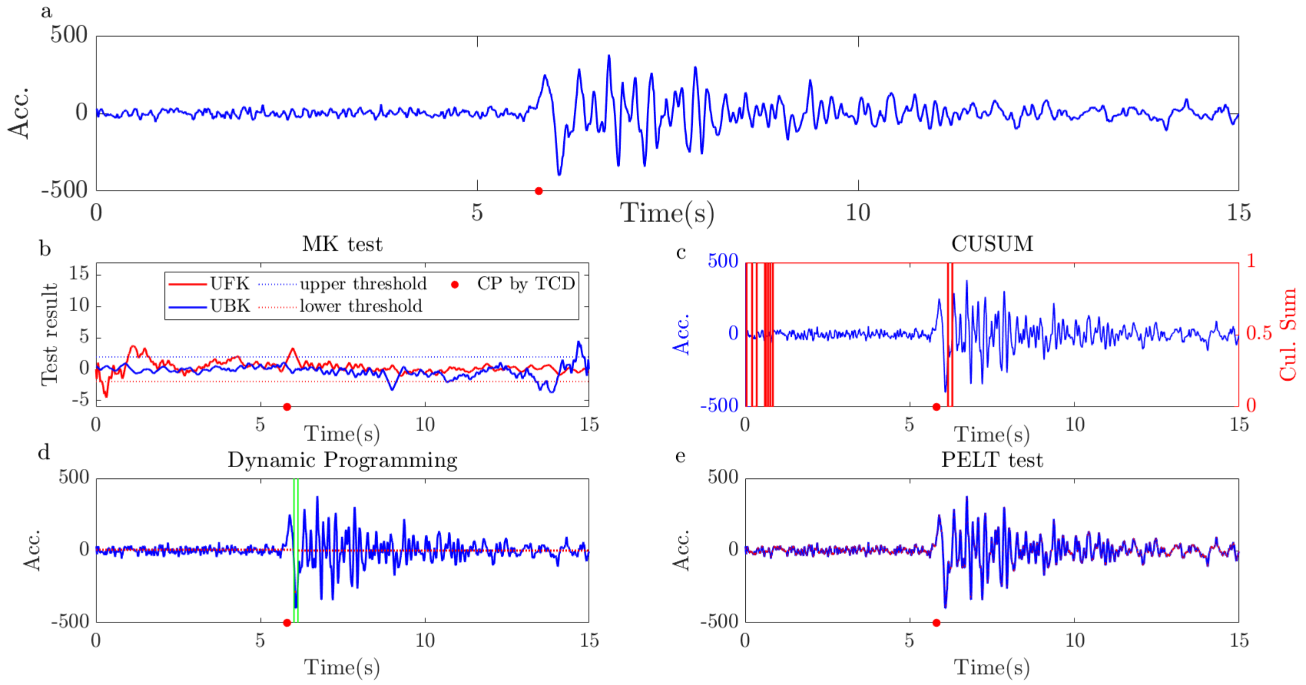


**Figure S11. The detection results, using the four representative statistical methods, respectively, on the earthquake strong motion dataset.** **a**. The original time series of the S60W acceleration, where the change point detected using the TCD approach is marked by the red dot. **b**. The results of using the Mann-Kendall test: No clear change points (the intersections of UFK and UBK between the thresholds) are detected. **c**. The results of using the CUSUM: The detected change points (red vertical lines) are later than the TCD-detected change point. **d**. The results of using the Dynamic Programming: The detected change points (the green vertical lines) are still later than the TCD-detected change point. The red dotted lines represent the mean values of the time series in each segment divided by these detected change points. **e**. The results of using the PELT test: No significant change points (green vertical lines) are detected.

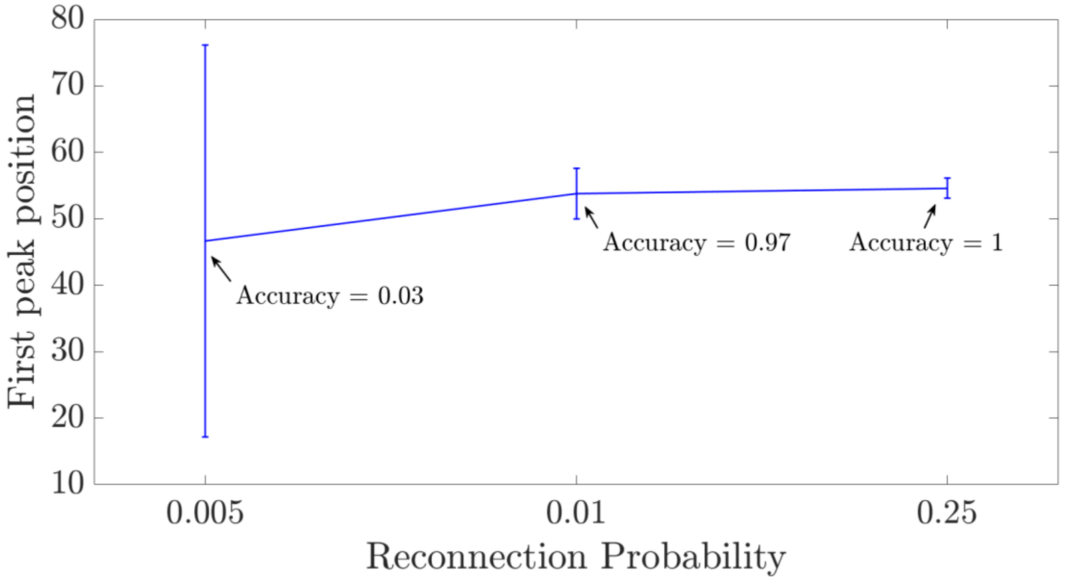


**Figure S12. The influence of *p*, the reconnection probability for the small-world network, on the detection accuracy of the change points in the LORENZ30 model (A7).** Here, the change point is set as $t = 50$. For each reconnection probability value, we run 500 independent simulations with random initial values. We simply record the loci where the prediction error reaches its maximum value. If it is located within the interval [51, 60], we regard this event as a successful detection. The detection accuracy for each reconnection probability is then defined as the proportion of successful detections among all the solid reconnections.

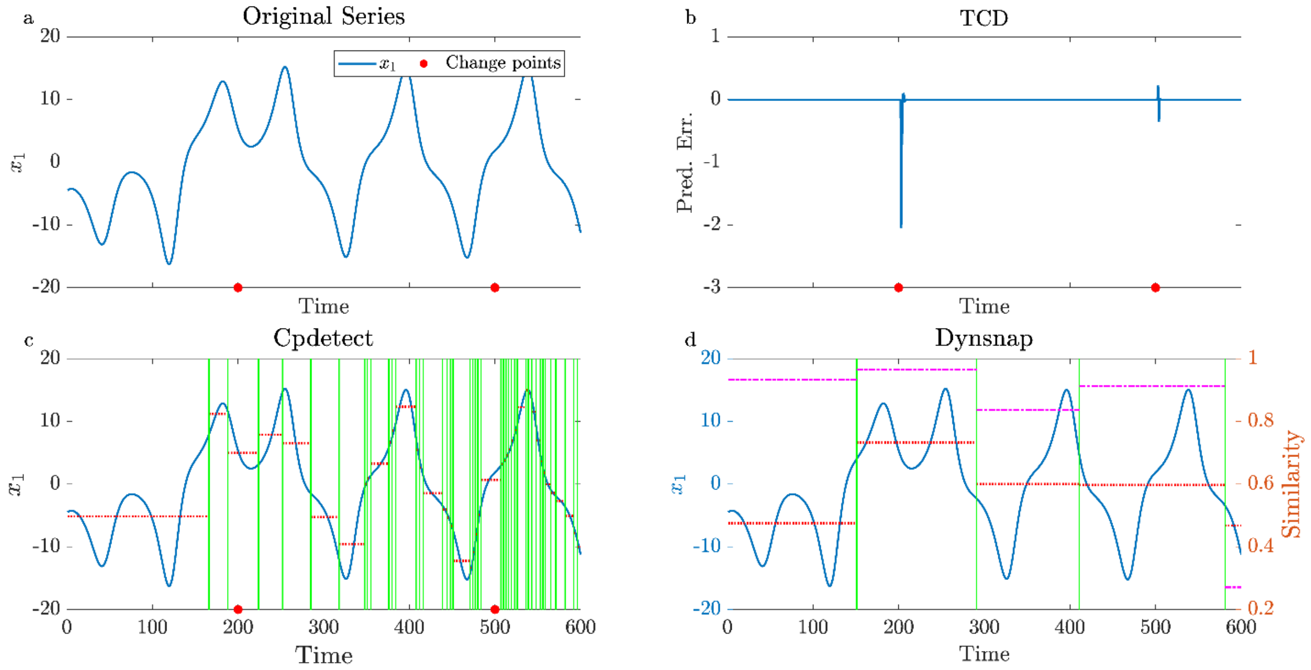


**Figure S13. The detection results obtained by using, respectively, TCD and two time-series segmentation methods on the data generated by the LORENZ15a system driven by events at non-uniform time intervals.** **a.** The original time series of $x_1$ in the system. The two change points, marked as red dots, are preset non-uniformly at $t_1 = 200$ and $t_2 = 500$, respectively. **b.** The detection results obtained by using our TCD approach. Clearly, our approach detects the two change points by measuring the prediction errors. **c.** The detection results obtained by using the Cpdetect algorithm. None of the detected change points (green solid lines) coincide with the true change points. Here, the red dotted lines represent the mean values of the time series in each segment divided by these detected change points. **d.** The detection results obtained by using the Dynsnap algorithm. Analogous to Cpdetect, Dynsnap fails to recognize any preset change points and the detected change points are of false-positive results. Here, the red dotted lines represent the mean values of the time series in each segment divided by these detected change points, and the magenta dot-dash lines represent the similarity measures within each segment.

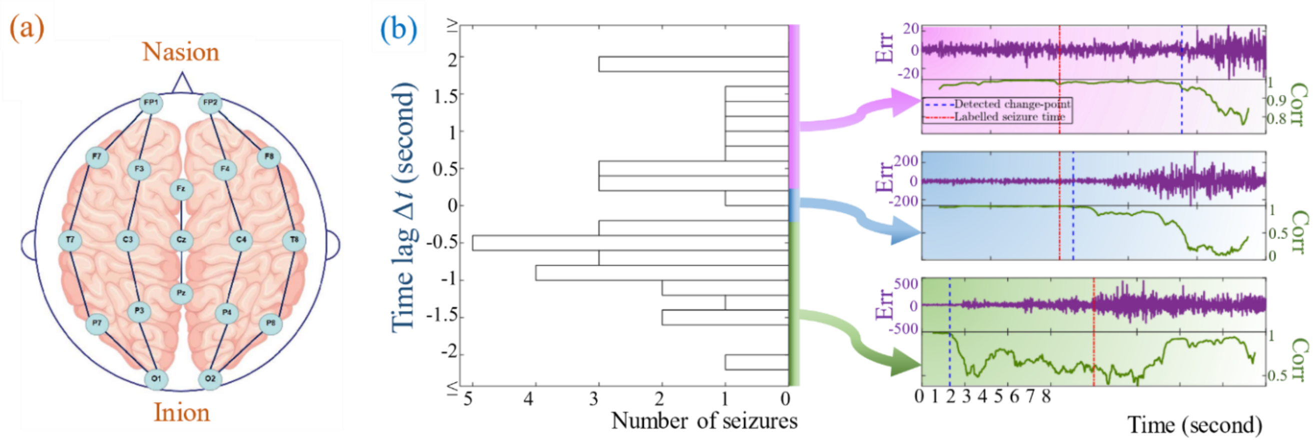


**Figure S14. Change-point detection for the EEG datasets.** **a.** The head distribution of the18-channel EEG leads used for collecting data in the database. **b.** The time-lag distribution of the detected seizures. The time-lag, Δt, stands for the difference between the detected change point using our approach and the seizure time labelled by the experts in the database. Particularly, $\Delta t<0$ indicates the realization of the seizure forecast, while $\Delta t\geqslant 0$ represents the seizure identification after but very close to the labelled time. On the right side of the panel **b**, displayed are the typical prediction errors (Err's, the violet curves) and the correlation coefficients (Corr's, the green curves), corresponding to three cases: $\Delta t<0$, $\Delta t\approx 0$ and $\Delta t>0$, respectively.

Here, based on the results in the literature, we present some analytical estimations for the algorithm of the Bayesian Online Changepoint Detection (BOCD). Specifically, we provide the asymptotic approximations of the distribution of run-length for the general case and for the case of the Gaussian distribution as well.

## S1.1 The concept of run-length

Assume that we have a series of time points, denoted by $\boldsymbol{x}_{1:T} = \{x_i\}_{i=1}^{T}$. In the BOCD test, we aim at estimating $r_t$, the run-length (RL) of each step, given the observations before the time $t\,(< T)$. As such, we estimate the RL's posterior distribution $p(r_t|\boldsymbol{x}_{1:t})$. As is introduced in the main text, the evolution of $r_t$ obeys the following rule:

$$r_t = \begin{cases} 0, & \text{if the change point appears at time } t, \\ r_{t-1} + 1, & \text{otherwise.} \end{cases}$$

Theoretically, when there is no change point before time $t$, $r_t$ is the most likely to be at the value $t$, so that $p(r_t = t|\boldsymbol{x}_{1:t}) > p(r_t = k|\boldsymbol{x}_{1:t})$ for any $0 \le k < t$. In other words, for some time $s$, if there exists a number $k^*$ with $0 \le k^* < s$ such that $p(r_{s+1} = s+1|\boldsymbol{x}_{1:s+1}) < p(r_{s+1} = k^*|\boldsymbol{x}_{1:s+1})$, it is highly likely that there exists a change point at time $s - k^*$. Without loss of generality for this problem, we simply compare the joint probabilities $p(r_{s+1} = s+1; \boldsymbol{x}_{1:s+1})$ and $p(r_{s+1} = 0; \boldsymbol{x}_{1:s})$ for judging the existence of a change point at time $s$. Here, $s$ is supposed to be the first potential change point in the time series. For the other case of multiple change points, the following approximations still hold by considering a direct time shift.

According to the Bayesian inference, the joint distribution $p(r_{s+1}, \boldsymbol{x}_{1:s})$ could be written recursively as [1]:

$$\begin{aligned} p(r_{s+1}, \boldsymbol{x}_{1:s+1}) &= \sum_{r_s} p(r_{s+1}, r_s, x_{s+1}, \boldsymbol{x}_{1:s}) \\ &= \sum_{r_s} p(r_{s+1}, x_{s+1}|r_s, \boldsymbol{x}_{1:s}) p(r_s, \boldsymbol{x}_{1:s}) \\ &= \sum_{r_s} \underbrace{p\left(x_{s+1}|r_s, \boldsymbol{x}^{(l)}\right)}_{\text{Predictive}} \underbrace{p(r_{s+1}|r_s)}_{\text{Prior}} \underbrace{p(r_s, \boldsymbol{x}_{1:s})}_{\text{Message}}, \#(\text{S1}) \end{aligned}$$

where $\boldsymbol{x}^{(l)} := \boldsymbol{x}_{s-l:s}$ is defined as the observations since the last change point, and the third equation holds because $r_s$ and $\boldsymbol{x}^{(l)}$ contain all the required information to predict $x_{s+1}$.

## S1.2 Run-length predictive from exponential family likelihoods

In the BOCD test, the observations $\{x_t\}$ are supposed to be i.i.d. from a distribution in the exponential family for each segment divided by the change points [1]. Given a finite number of sufficient statistics, the exponential family likelihood is written as:

$$p(\boldsymbol{x}|\boldsymbol{\eta}) = h(\boldsymbol{x}) g(\boldsymbol{\eta}) \exp\left(\boldsymbol{\eta}^{\mathrm{T}} u(\boldsymbol{x})\right), \#(\text{S2})$$

where $\boldsymbol{\eta}$ denotes the natural parameter, $h(\boldsymbol{x})$ is called the underlying measure, $u(\boldsymbol{x})$ is the sufficient statistic of the data series, and $g(\boldsymbol{\eta})$ is a normalizer which ensures

$$g(\boldsymbol{\eta}) \int h(\boldsymbol{x}) \exp\left(\boldsymbol{\eta}^{\mathrm{T}} u(\boldsymbol{x})\right) = 1.$$

Exponential family contains a large number of familiar probabilistic distributions. Just to name a few, the Bernoulli distribution, the Poisson distribution, the Gamma distribution, and the Gaussian distribution.

Regarding the estimation of the natural parameters through the Bayesian Inference, conjugacy is an important and useful property. Suppose that a distribution in the exponential family is equipped with hyperparameters $v$ and $\chi$, and the conjugate prior of the hyperparameters is written as:

$$p(\boldsymbol{\eta}|v, \boldsymbol{\chi}) = f(v, \boldsymbol{\chi}) g(\boldsymbol{\eta})^{v} \exp(\boldsymbol{\eta}^{\mathrm{T}} \boldsymbol{\chi}), \#(\text{S3})$$

where $f(v, \boldsymbol{\chi})$ depends on the form of the distribution. In Ref. [2], it has been proved that

$$p(\boldsymbol{\eta}|\boldsymbol{x}_{1:s}, v, \boldsymbol{\chi}) \propto g(\boldsymbol{\eta})^{s+v} \exp\left(\boldsymbol{\eta}^{\mathrm{T}}\left(\sum_{i=1}^{s} u(x_i) + \boldsymbol{\chi}\right)\right). \#(\mathrm{S4})$$

The above is also an exponential-family form, with the hyperparameters $v' = v + s$, $\boldsymbol{\chi}' = \boldsymbol{\chi} + \sum_{i=1}^{s} u(x_i)$. This implies that we can estimate the RL predictive using updated parameters recursively.

For the initial conditions, we set $v_t^0 = v_{prior}$ and $\boldsymbol{\chi}_t^0 = \boldsymbol{\chi}_{prior}$ as our prior knowledge of the hyperparameters. And the updating process could be described as $v_t^l = v_{t-1}^{l-1} + 1$, $\boldsymbol{\chi}_t^l = \boldsymbol{\chi}_{t-1}^{l-1} + u(x_t)$, where $v_t^l$ and $\boldsymbol{\chi}_t^l$ denote the hyperparameters for each run-length value $r_t = l$ at time $t$.

## S1.3 Change point prior

The change point prior quantifies the probability that the change point occurs at $\tau$ provided that there has been no change point before $\tau$. To address this, the hazard function $H(\tau) = P(T > \tau)$ is introduced to describe the conditional prior on the change point:

$$p(r_t|r_{t-1}) = \begin{cases} H(r_{t-1} + 1), & \text{if } r_t = 0, \\ 1 - H(r_{t-1} + 1), & \text{if } r_t = r_{t-1} + 1, \end{cases} \#(\mathrm{S5})$$

where $T$ is a discrete and nonnegative random variable for the current RL. Usually, $T$ is supposed to follow a geometric distribution with a success probability $H$, and then we have $H(\tau) = H$ [3].

## S1.4 Approximations of the joint probabilities

Using the notations and Eqs. (S1)-(S5) introduced above, we, respectively, have

$$\begin{aligned} p(r_{s+1} = s + 1; \boldsymbol{x}_{1:s+1}) &= p(x_{s+1}|r_s = s, \boldsymbol{x}_{1:s}) p(r_{s+1} = r_s + 1|r_s) p(r_s = s; \boldsymbol{x}_{1:s}) \\ &= (1 - H) p(x_{s+1}|\boldsymbol{x}_{1:s}) p(r_s = s; \boldsymbol{x}_{1:s}) \\ &= (1 - H) p(x_{s+1}|v_s^s, \boldsymbol{\chi}_s^s) p(r_s = s; \boldsymbol{x}_{1:s}) \\ &= (1 - H)^s \prod_{i=1}^{s} p\left(x_{i+1}|v_i^i, \boldsymbol{\chi}_i^i\right) p(r_1 = 1; x_1) \\ &= (1 - H)^{s+1} \prod_{i=1}^{s} p\left( x_{i+1} \middle| v_{prior} + i, \boldsymbol{\chi}_{prior} + \sum_{j=1}^{i} u(x_j) \right), \#(\mathrm{S7}) \end{aligned}$$

and

$$\begin{aligned} p(r_{s+1} = 0; \boldsymbol{x}_{1:s+1}) &= \sum_{r_s = r} p\left(x_{s+1}|r_s, \boldsymbol{x}^{(r)}\right) p(r_{s+1} = 0|r_s)\, p(r_s; \boldsymbol{x}_{1:s}) \\ &= H \sum_{r=0}^{s} p(x_{s+1}|v_s^r, \boldsymbol{\chi}_s^r)\, p(r_s = r; \boldsymbol{x}_{1:s}). \#(\mathrm{S8}) \end{aligned}$$

The calculation for $p(r_{s+1} = s + 1; \boldsymbol{x}_{1:s+1})$ is really straightforward because there is only one feasible path from $r_0 = 0$ to $r_s = s$. However, the calculation for $p(r_{s+1} = 0; \boldsymbol{x}_{1:s+1})$ is more sophisticated. For a one-step detection, i.e., assuming $r_s \approx 1$, we have:

$$p(r_{s+1} = s + 1; \boldsymbol{x}_{1:s+1}) : p(r_{s+1} = 0; \boldsymbol{x}_{1:s+1}) = 1 - H : H. \#(\mathrm{S9})$$

For multi-step detections, this ratio relies on the certain probabilistic distribution that $\{x_t\}$ follows. For example, suppose that the observations follow the Gaussian distribution with a consistent variance $\sigma^2 = 1$ but a changing mean $\mu_t$. We let $\lambda = \lambda_{\mathrm{prior}} = 1/\sigma^2$, and the posterior predictive for this model becomes:

$$p(x_{t+1}|\boldsymbol{x}_{1:t}) = \mathcal{N}\left(x_{t+1} \mid \mu_t, 1 + \frac{1}{\lambda_t}\right), \#(\mathrm{S10})$$

where

$$\mu_t = \frac{\mu_{\text{prior}} + \sum_{i=1}^{t} x_i}{t+1} \quad \text{and} \quad \lambda_t = \lambda_{\text{prior}} + t.$$

Therefore, the updated parameters in BOCD could be written as

$$\mu_t^r = \frac{t\mu_{t-1}^r + x_t}{t+1} \quad \text{and} \quad \lambda_t^r = 1 + \lambda_{t-1}^r.$$

Here, we briefly list the results of the first several joint probabilities:

$$p(r_1 = 1; x_1) = 1 - H,$$

$$p(r_1 = 0; x_1) = H,$$

$$p(r_2 = 2; \boldsymbol{x}_{1:2}) = (1-H)^2 \frac{\lambda_1^1}{\sqrt{2\pi}} \exp\left(-\frac{\lambda_1^1 (x_2 - \mu_1^1)^2}{2}\right),$$

$$p(r_2 = 0; \boldsymbol{x}_{1:2}) = H(1-H) \frac{\lambda_1^1}{\sqrt{2\pi}} \exp\left(-\frac{\lambda_1^1 (x_2 - \mu_1^1)^2}{2}\right) + H^2 \frac{\lambda_1^0}{\sqrt{2\pi}} \exp\left(-\frac{\lambda_1^0 (x_2 - \mu_1^0)^2}{2}\right),$$

$$p(r_3 = 3; \boldsymbol{x}_{1:3}) = (1-H)^3 \frac{\lambda_1^1 \lambda_2^2}{(\sqrt{2\pi})^2} \exp\left(-\frac{\lambda_1^1 (x_2 - \mu_1^1)^2 + \lambda_2^2 (x_3 - \mu_2^2)^2}{2}\right),$$

$$p(r_3 = 0; \boldsymbol{x}_{1:3}) = H(1-H)^2 \frac{\lambda_1^1 \lambda_2^2}{(\sqrt{2\pi})^2} \exp\left(-\frac{\lambda_1^1 (x_2 - \mu_1^1)^2 + \lambda_2^2 (x_3 - \mu_2^2)^2}{2}\right)$$

$$+H^2(1-H) \frac{\lambda_1^0 \lambda_2^1}{(\sqrt{2\pi})^2} \exp\left(-\frac{\lambda_1^0 (x_2 - \mu_1^0)^2 + \lambda_2^1 (x_3 - \mu_2^1)^2}{2}\right)$$

$$+H^2(1-H) \frac{\lambda_1^1 \lambda_2^0}{(\sqrt{2\pi})^2} \exp\left(-\frac{\lambda_1^1 (x_2 - \mu_1^1)^2 + \lambda_2^0 (x_3 - \mu_2^0)^2}{2}\right)$$

$$+H^3 \frac{\lambda_1^0 \lambda_2^0}{(\sqrt{2\pi})^2} \exp\left(-\frac{\lambda_1^0 (x_2 - \mu_1^0)^2 + \lambda_2^0 (x_3 - \mu_2^0)^2}{2}\right). \#(S11)$$

For most of the real-world data series (including all the datasets in the main text), $H$ is set as a very small value, i.e., $H \ll 1$. Therefore, according to the above calculations, the ratio $p(r_{s+1} = s+1; \boldsymbol{x}_{1:s+1}) : p(r_{s+1} = 0; \boldsymbol{x}_{1:s+1})$ is roughly determined by $(1-H)^s : H$. In other words, the probability that the current time point is not a change point decreases exponentially along the time evolution until a change point is detected. In practice, many of the values of the posterior run-length are so small that one may force any values less than a certain threshold to be zero, which can significantly release the computational burdens [4].

# Appendix

## A1. Coupled Lorenz systems with temporal structures

We consider in the main text the $N$-dimensional coupled Lorenz systems, whose individual equation is described by

$$\begin{cases} \dfrac{\mathrm{d}x_i}{\mathrm{d}t} = \sigma(x_i - y_i) + C \cdot \boldsymbol{A}_k \boldsymbol{x}, \\ \dfrac{\mathrm{d}y_i}{\mathrm{d}t} = \rho x_i - y_i - x_i z_i, \\ \dfrac{\mathrm{d}z_i}{\mathrm{d}t} = x_i y_i - \beta z_i, \end{cases}$$

$$i = 1,2, \ldots, N. \qquad \text{(A1)}$$

where $\sigma = 10$, $\rho = 28$, $\beta = 8/3$ are typical parameters for generating chaotic dynamics, $\boldsymbol{A}_k$ denotes the adjacent matrix of the network, and the coupling strength is set as $C = 0.1$. Additionally, $\boldsymbol{x} = [x_1, x_2, \ldots, x_N]^{\mathrm{T}}$ is a vector containing all the first components of the individual Lorenz system [5]. For the results in Figs. 1(a)-(c) of the main text, we take $N = 20$ (i.e., the coupled systems become the 60-dimensional LORENZ60 model), and configure the couplings in (A1) in a manner that the adjacent matrix $\boldsymbol{A}_k = \{a_{kl}\}$ takes its matrix value from the set $\{\boldsymbol{A}_1, \boldsymbol{A}_2, \boldsymbol{A}_3\}$ in some time duration. As particularly shown in Fig. S1, the matrices in the set, representing coupling networks, are constructed as follows: (1) obtain the matrix $\boldsymbol{A}_1$ by connecting the neighboring nodes in the network, i.e., connect $x_i$ with $x_{i+1}$ and $x_{i-1}$, and denoting by $x_{1-1} := x_N$ and $x_{N+1} := x_1$ for making the system closed, and (2) obtain the matrices $\boldsymbol{A}_2$ and $\boldsymbol{A}_3$ independently such that, for $i, j = 1,2, \ldots, N$, $i \neq j$, randomly connect $x_i$ with $x_j$ unidirectionally with a probability of $p = 0.1$ (i.e., $a_{ij} = 1$), and $a_{ii}$ is always set as $0$. Furthermore, we set the adjacent matrix to be temporally switching in the following manner:

$$\boldsymbol{A}_k = \begin{cases} \boldsymbol{A}_1, 0 \le t < t_1 \\ \boldsymbol{A}_2, t_1 \le t < t_2 \\ \boldsymbol{A}_3, t \ge t_2 \end{cases} \qquad \text{(A2)}$$

For the results in the main text on the robustness against noise, the efficacy or/and the sensitivity tests on different configurations of networks and change-point frequency, and the comparison study with the other representative statistical methods, different sorts of 15-dimensional LORENZ15 models are used. With an integration step length $\Delta t = 0.01$, we solve the systems numerically by the standard Euler method and then produce the dataset. Here, we list the LORENZ15 models in different coupling sorts as follows. The LORENZ15a model is written as:

$$\begin{cases} \dfrac{\mathrm{d}x_i}{\mathrm{d}t} = \sigma(x_i - y_i) + C \cdot \boldsymbol{B}_k \boldsymbol{x}, \\ \dfrac{\mathrm{d}y_i}{\mathrm{d}t} = \rho x_i - y_i - x_i z_i, \\ \dfrac{\mathrm{d}z_i}{\mathrm{d}t} = x_i y_i - \beta z_i, \end{cases}$$

$$i = 1,2, \ldots, 5. \qquad \text{(A3)}$$

where the $5 \times 5$ adjacent matrix $\boldsymbol{B}_k$ takes its value from the set $\{\boldsymbol{B}_1, \boldsymbol{B}_2, \boldsymbol{B}_3\}$ in a switching manner, as illustrated in Fig. S1. In the study of the robustness against noise, we take into account two types of additive noises are: the dynamical noise (i.e., the white noise is added to the vector fields) and the observational noise (i.e., the white noise is added to the observational time series directly). For the first type, we introduce the noise into the LORENZ15a model (A3) as:

$$\begin{cases} \dfrac{\mathrm{d}x_i}{\mathrm{d}t} = \sigma(x_i - y_i) + C \cdot \boldsymbol{B}_k \boldsymbol{x} + V W_t, \\ \dfrac{\mathrm{d}y_i}{\mathrm{d}t} = \rho x_i - y_i - x_i z_i, \\ \dfrac{\mathrm{d}z_i}{\mathrm{d}t} = x_i y_i - \beta z_i, \end{cases}$$

$$i = 1,2, \ldots, 5. \qquad \text{(A3}'\text{)}$$

where $W_t$ represents the standard Brownian motion and $V$ is the noise strength set at different levels. For the latter type,

we simply add the white noise with different noise strengths directly to the synthetized data produced by model (A3).

The LORENZ15b model is written as:

$$\begin{cases} \dfrac{\mathrm{d}x_i}{\mathrm{d}t} = \sigma(x_i - y_i) + C \cdot \boldsymbol{B}_1\boldsymbol{x}, \\ \dfrac{\mathrm{d}y_i}{\mathrm{d}t} = \rho x_i - y_i - x_i z_i, \\ \dfrac{\mathrm{d}z_i}{\mathrm{d}t} = x_i y_i - \beta z_i, \end{cases}$$

$$i = 1,2,\ldots,5. \qquad \text{(A4)}$$

Here, we concentrate on the change-point detection thresholds for the following parameters: $\sigma$, $\rho$, $\beta$, and $C$. Specifically, the parameter values change from the default value to a new value at a preset change point. The difference between the new value and the default value is decreased until the change point cannot be detected using the TCD approach and the threshold difference of each parameter is recorded. Furthermore, we validate the impact of the position of the change point on the detection problem. The default change point is set at $t = 2000$, after which the value of parameter $\sigma$ is changed from 10 to 10.2. Then we alter its position and calculate the time lag between the detected change point and the preset change point. All the corresponding results are listed in Table S2 and in Fig. S9 as well.

The LORENZ15c model, whose coupling structures *change gradually in a linear manner*, is written as:

$$\begin{cases} \dfrac{\mathrm{d}x_i}{\mathrm{d}t} = \sigma(x_i - y_i) + C \cdot \boldsymbol{B}(t)\boldsymbol{x}, \\ \dfrac{\mathrm{d}y_i}{\mathrm{d}t} = \rho x_i - y_i - x_i z_i, \\ \dfrac{\mathrm{d}z_i}{\mathrm{d}t} = x_i y_i - \beta z_i, \end{cases}$$

$$i = 1,2,\ldots,5. \qquad \text{(A5)}$$

Here, $\boldsymbol{B}(t)$, a piecewise function, is fixed as $\boldsymbol{B}_2$ for $t < t_1$ and as $\boldsymbol{B}_3$ for $t > t_2$. And, when $t_1 < t < t_2$, $\boldsymbol{B}(t)$ linearly changes from $\boldsymbol{B}_2$ to $\boldsymbol{B}_3$.

$$\begin{cases} \dfrac{\mathrm{d}x_i}{\mathrm{d}t} = \sigma(x_i - y_i) + C \cdot \boldsymbol{B}_1\boldsymbol{x}, \\ \dfrac{\mathrm{d}y_i}{\mathrm{d}t} = \rho x_i - y_i - x_i z_i, \\ \dfrac{\mathrm{d}z_i}{\mathrm{d}t} = x_i y_i - \beta z_i, \end{cases}$$

$$i = 1,2,\ldots,5. \qquad \text{(A6)}$$

To validate efficacy of our approach in detection of change points emergent within a high frequency, we consider LORENZ15d model as:

Here, $\tilde{\rho}$ is a time-varying parameter orchestrating its value switching between $\rho_1$ and $\rho_2$ at each change point, where the values of $\rho_1$ and $\rho_2$ are picked from the set $\{27.1, 27.4, 27.7, 28.3, 28.6, 28.9\}$. Thus, totally 15 different group of switching structures could be used. For our approach, all the groups are applied and repeated for 30 times using 30 different 3-dimensional randomly-chosen tuples. And for the other two methods, the MK and the BOCD tests, one group is used, simply because these statistical methods perform poorly in detection of change points even the switching frequency is not that high.

To investigate on the influence of network structures on our TCD approach, we apply a 30-dimensional LORENZ30 model, which is described as:

$$\begin{cases} \dfrac{\mathrm{d}x_i}{\mathrm{d}t} = \sigma(x_i - y_i) + C \cdot \boldsymbol{C}_k\boldsymbol{x}, \\ \dfrac{\mathrm{d}y_i}{\mathrm{d}t} = \rho x_i - y_i - x_i z_i, \\ \dfrac{\mathrm{d}z_i}{\mathrm{d}t} = x_i y_i - \beta z_i, \end{cases}$$

$$i = 1,2,\ldots,10. \quad \text{(A7)}$$

where $\boldsymbol{C}_k = \boldsymbol{C_1}$ for $t < t_1$ and $\boldsymbol{C}_k = \boldsymbol{C_2}$ for $t \geq t_1$. The construction of $\boldsymbol{C_k}$ follows the same procedure as that of $\boldsymbol{A_k}$ except for their difference on dimensionalities. In addition to the standard Euler method, we apply the fourth-order Runge-Kutta method (RK4) to solve model (A7), which yields the results that are the same as those using the Euler method with sufficiently small step size.

## A2. The biochemical oscillator with alterable removal rate

A biochemical system containing two coupled allosteric enzymes [6] is used to demonstrate our proposed approach in the main text. In such a system, the synthesis of the two products, $P_1$ and $P_2$, from the precursor, $S$, is catalyzed by the two enzymes, and the enzymes form the two positive feedback loops coupled in the chemical reaction procedure, which can result in various modes of dynamical behaviors. The system is governed by the following mathematical equations, in which the enzymes obey the model proposed by Monod *et al.* [7]:

$$\begin{cases} \dfrac{\mathrm{d}x}{\mathrm{d}t} = \dfrac{v}{K_{m1}} - \sigma_1\varphi, \\ \dfrac{\mathrm{d}y_1}{\mathrm{d}t} = q_1\sigma_1\varphi - \sigma_2\psi, \\ \dfrac{\mathrm{d}y_2}{\mathrm{d}t} = q_2\sigma_2\psi - k_s P_2, \end{cases} \#\text{(A8)}$$

where the positive feedback terms are defined, respectively, by

$$\varphi = \frac{x(1+x)(1+y_1)^2}{L_1 + (1+x)^2(1+y_1)^2},$$
$$\psi = \frac{y_1(1+dy_1)(1+y_2)^2}{L_2 + (1+dy_1)^2(1+y_2)^2}. \#\text{(A9)}$$

Here, $x$, $y_1$ and $y_2$ denote the concentrations of $S$, $P_1$ and $P_2$, respectively. We set $v/K_{m1} = 0.45$ sec$^{-1}$, $\sigma_1 = \sigma_2 = 10$ sec$^{-1}$, $q_1 = 50$, $q_2 = 0.02$, $L_1 = 5 \times 10^8$, $L_2 = 100$, and $d = 0$. The parameter values of the removal rate $k_s$ for $P_2$ is set to be 1.97 sec$^{-1}$ or 2.00 sec$^{-1}$ (separated by a change point at $t = 500$) so as to demonstrate our approach of change-point detection. In the study, a time series containing 1000 time points is used. Before $t = 0$ the attractor has shown a stabilized limit cycle. The variables were pre-treated by taking into account the time-delayed coordinates so as to render the observable system having sufficiently high dimensionality. Here, we use the time-delayed coordinates up to 2 for each variable in the model, so that the observable system becomes $\boldsymbol{x}(t) = [x(t), x(t-1), x(t-2), y_1(t), y_1(t-1), y_1(t-2), y_2(t), y_2(t-1), y_2(t-2)]^{\mathrm{T}}$.

## A3. The earthquake strong motion dataset

The New Zealand Strong Motion Database consists of strong motion records from the Palmerston North Telephone Exchange station [8,9]. For measuring strong shaking with damaging earthquakes, strong-motion accelerometers are located in major centers of population, near significant faults, or in different types of building structures. The three components of the multivariate strong motion series are the three orthogonal directions namely N30W, S60W, and UP. The time series is recorded at 100Hz and comprises of 1500 observations that are illustrated in Fig S2.

Our TCD method aims to detect dynamic change points on the acceleration time series of S60W direction. To achieve this goal, we take the time-delayed coordinates up to 5 for each direction and form an 18-dimensional system. Specifically, the system could be written as:

$$x_1(t+1) = F(\boldsymbol{x}_{1t}, \boldsymbol{x}_{2t}, \boldsymbol{x}_{3t}), \quad \text{(A10)}$$

where $x_1(t), x_2(t), x_3(t)$ represent, respectively, the acceleration (in $m/s^2$) at time $t$ at N30W, S60W and UP, and each

$$\boldsymbol{x}_{jt} = \left[x_j(t), x_j(t-1), x_j(t-2), x_j(t-3), x_j(t-4), x_j(t-5)\right]^{\mathrm{T}} \#\text{(A11)}$$

is a vector using the time-delayed coordinates with $j = 1,2,3$.

## A4. The Greenland dataset

The Greenland dataset consists of the dated isotope concentration records from the Greenland GRIP, GISP2, and NGRIP ice cores [10]. The synchronization of the ice cores was previously presented to provide a detailed comparison among different ice cores [11-14]. Specifically, we consider the $\delta^{18}$O records of three ice cores, defined by

$$\delta^{18}\mathrm{O} = \left( \frac{\left(\frac{^{18}\mathrm{O}}{^{16}\mathrm{O}}\right)_{\mathrm{sample}}}{\left(\frac{^{18}\mathrm{O}}{^{16}\mathrm{O}}\right)_{\mathrm{standard}}} - 1 \right) * 1000\ ‰\,, \tag{A12}$$

where the standard mean ocean water that has a known isotopic composition. Considering the fact that the saturated vapor pressure of $H_2{}^{18}O$ is subtly lower than that of $H_2{}^{16}O$, the variations in the relative concentration of $^{18}$O can represent the change in the temperature of the ice core through the geological time.

In this dataset, each data point represents a 20-year-mean of $\delta^{18}$O concentration recorded from 50000 to 40800 years b2k (years before 2000 CE) on the GICC05 timescale (Fig. S3) [13,14]. Since interactions between the climate events and isotope concentration may take time, we consider the time-delayed coordinates up to 5 for the records from each ice core. Similarly, the system could be written as:

$$x_1(t+1) = F(\boldsymbol{x}_{1t}, \boldsymbol{x}_{2t}, \boldsymbol{x}_{3t})\,, \tag{A13}$$

where $x_1(t), x_2(t),$ and $x_3(t)$ represent the $\delta^{18}$O concentration (in ‰) at time $t$, respectively, of NGRIP, GRIP, and GISP2, and each $\boldsymbol{x}_{jt} = \left[x_j(t), x_j(t-1), x_j(t-2), x_j(t-3), x_j(t-4), x_j(t-5)\right]^{\mathrm{T}}$ for $j = 1,2,3$.

## A5. The financial market dataset

We consider a financial market system in the main text that contains the closing price time series of three stocks sampled every trading day from Jan 2008 to Dec 2009. The three stocks are modeled as a multivariate time series, including Intel Corporation（Integrated Electronics Corporation, INTC), Cisco Systems Inc (CSCO), and the Victory RS Small Cap Equity Fund Class A (GPSCX). The raw data are collected at http://finance.yahoo.com, and are depicted in Fig. S5.

In this the main context, we consider the impact of global economic crisis on the closing price of CSCO. Similar to the above real-world datasets, we use a 15-dimensional system that consists of the time-delayed coordinates up to 5 for the records from each stock.

## A6. The EEG dataset

The Scalp Electroencephalogram (Scalp EEG) data set, provided by *PhysioNet*, is composed of the EEG recordings pediatric subjects with intractable seizures collected at the Children's Hospital Boston [15,16]. Recordings, grouped into 23 cases, were collected from 22 subjects (5 males, ages 3–22; and 17 females, ages 1.5–19). Here, we validate our method on randomly-picked 2 males and 6 females by analyzing their EEG records with seizure. In all, 40 out of 129 files that contain one or more seizures were validated.

All signals we used in the main text were sampled at 256 samples per second with 16-bit resolution, and each EEG file was recorded using an 18-channel, 10-20 bipolar montage. The beginning and the end of each epileptic seizure is annotated in the database. The whole database as well as its descriptions is available at https://archive.physionet.org/pn6/chbmit/ .

The occurrence of an epileptic seizure could be considered as an instant where the predictability changes suddenly and dramatically. To detect such occurrence, we use a window containing 25 time points as a training set, make one-step prediction on the target channel along the axis of time, and focus on the prediction fidelity changing with time. We calculate the shifting-window correlation coefficient between the one-step prediction values and the real values. Once we quantitatively locate a dramatic change on the correlation coefficient with respect to either channel of the 18 channels, we confirm the occurrence of the seizure. To show the effectiveness of our approach, we compare the change points that we detect and the seizure time that the experts labelled for the original database. As is shown in Fig. S14, among the 42 samples in the database, we are able to detect 36 seizures. Indeed, among the detected seizures, 21 are detected in front of the labelled seizure time, which suggests that some structures dominating the brain dynamics are likely to change before the epileptic seizure is observed. This also reveals that our model-free approach could be potentially applicable to the early warning of seizure occurrence. There are still some detected seizures that are after but very close to the labelled time, as shown in Fig. S14b. This is also acceptable and helpful for efficiently and automatically realizing seizure diagnoses because conventional identification in clinics usually require labor and time cost for dealing with a huge amount of time series containing separately distributed seizures events.

## A7. Parameters

All the parameters in making one-step predictions using our TCD approach are summarized in Table S1.

**REFERENCES**

[1] Adams, R. P. & MacKay, D. J. C Bayesian online changepoint detection. Preprint at https://arXiv.org/abs/0710.3742 (2007).

[2] Murphy, K. P. The Exponential Family. http://gregorygundersen.com/blog/2019/03/19/exponential-family (2019).

[3] Forbes, C., Evans, M., Hastings, N. & Peacock, B. Statistical Distributions. Fourth Edition, *John Wiley & Sons, Hoboken* (2011).

[4] Byrd, M., Nghiem, L., & Cao, J. Lagged exact Bayesian online changepoint detection with parameter estimation. Preprint at https://arxiv.org/abs/1710.03276 (2017)

[5] Watts, D. J. & Strogatz, S. H. Collective dynamics of small world networks. *Nature* **393**, 440-442 (1998).

[6] Decroly, O. & Goldbeter, A. Birhythmicity, chaos, and other patterns of temporal self-organization in a multiply regulated biochemical system. *Proc. Natl. Acad. Sci. USA* **79**, 6917-21 (1982).

[7] Monod, J., Wyman, J. & Changeux, J. P. On the Nature of Allosteric Transitions: A Plausible Model. *J. Mol. Biol* **12**, 88-118 (1965).

[8] Van Houtte, C., Bannister, S., Holden, C., Bourguignon, S. & McVerry, G., The New Zealand Strong Motion Database, *Bulletin of the New Zealand Society for Earthquake Engineering*, in press (2017).

[9] Kaiser, A., Van Houtte, C., Perrin, N., Wotherspoon, L. & McVerry, G., Site characterisation of GeoNet stations for the New Zealand strong motion database, *Bulletin of the New Zealand Society for Earthquake Engineering*, in press (2017).

[10] Rasmussen, S. O., et al. A stratigraphic framework for abrupt climatic changes during the Last Glacial period based on three synchronized Greenland ice-core records: refining and extending the INTIMATE event stratigraphy. *Quat. Sci. Rev.* 4th INTIMATE special issue (2014).

[11] Seierstad, I. K., et al. Consistently dated records from the Greenland GRIP, GISP2 and NGRIP ice cores for the past 104ka reveal regional millennial-scale $\delta^{18}O$ gradients with possible Heinrich event imprint. *Quat. Sci. Rev.* **106**:29-46 (2014).

[12] Blockley, S. P. E., et al. Synchronization of paleoenvironmental records over the last 60,000 years, and an extended INTIMATE event stratigraphy to 48,000 b2k. *Quat. Sci. Rev.* **36**:2-10 (2012).

[13] Andersen, K. K., et al. The Greenland ice core chronology 2005, 15-42 ka. part 1: constructing the time scale. *Quat. Sci. Rev.* **25**(23):3246-3257 (2006).

[14] Svensson, A., et al. A 60 000 year Greenland stratigraphic ice core chronology. *Climate of the Past* **4**:47-57 (2008).

[15] Daniel L. E. and Vijay S. P. Bayesian Detection of Intensity Changes in Single Molecule and Molecular Dynamics

Trajectories. *J. Phys. Chem. B* **114**(1): 280–292 (2010).
[16] Darst, R., Granell, C., Arenas, A., et al. Detection of timescales in evolving complex systems. *Sci Rep* **6**: 39713 (2016).
